# Involvement of the choroid plexus in Alzheimer’s disease pathophysiology: findings from mouse and human proteomic studies

**DOI:** 10.1186/s12987-024-00555-3

**Published:** 2024-07-18

**Authors:** Aurore Delvenne, Charysse Vandendriessche, Johan Gobom, Marlies Burgelman, Pieter Dujardin, Clint De Nolf, Betty M. Tijms, Charlotte E. Teunissen, Suzanne E. Schindler, Frans Verhey, Inez Ramakers, Pablo Martinez-Lage, Mikel Tainta, Rik Vandenberghe, Jolien Schaeverbeke, Sebastiaan Engelborghs, Ellen De Roeck, Julius Popp, Gwendoline Peyratout, Magda Tsolaki, Yvonne Freund-Levi, Simon Lovestone, Johannes Streffer, Lars Bertram, Kaj Blennow, Henrik Zetterberg, Pieter Jelle Visser, Roosmarijn E. Vandenbroucke, Stephanie J. B. Vos

**Affiliations:** 1 Department of Psychiatry and Neuropsychology, Alzheimer Centrum Limburg, School for Mental Health and Neuroscience, Maastricht University, P.O. Box 616, Maastricht, 6200 MD The Netherlands; 2 VIB Center for Inflammation Research, VIB, Ghent, Belgium; 3 Department of Biomedical Molecular Biology, Ghent University, Ghent, Belgium; 4 Clinical Neurochemistry Laboratory, Sahlgrenska University Hospital, Mölndal, Sweden; 5 Department of Psychiatry and Neurochemistry, Institute of Neuroscience and Physiology, Sahlgrenska Academy at the University of Gothenburg, Mölndal, Sweden; 6 Department of Internal Medicine and Pediatrics, Ghent University, Ghent, Belgium; 7 Alzheimer Center Amsterdam, Department of Neurology, Amsterdam Neuroscience, Vrije Universiteit Amsterdam, Amsterdam UMC, Amsterdam, The Netherlands; 8 Neurochemistry Laboratory, Department of Clinical Chemistry, Amsterdam University Medical Centers (AUMC), Amsterdam Neuroscience, Amsterdam, Netherlands; 9 Department of Neurology, Washington University School of Medicine, St. Louis, USA; 10 Knight Alzheimer’s Disease Research Center, Washington University School of Medicine, St. Louis, USA; 11 Fundación CITA-Alzhéimer Fundazioa, San Sebastian, Spain; 12 Neurology Service, University Hospitals Leuven, Louvain, Belgium; 13 Laboratory for Cognitive Neurology, Department of Neurosciences, KU Leuven, Louvain, Belgium; 14 Reference Center for Biological Markers of Dementia (BIODEM), Department of Biomedical Sciences, University of Antwerp, Antwerp, Belgium; 15 Department of Neurology and Bru-BRAIN, Universitair Ziekenhuis Brussel, Brussels, Belgium; 16 NEUR Research Group, Center for Neurosciences (C4N), Vrije Universiteit Brussel, Brussels, Belgium; 17 Department of Neurology and Memory Clinic, Hospital Network Antwerp (ZNA) Middelheim and Hoge Beuken, Antwerp, Belgium; 18 Old Age Psychiatry, University Hospital Lausanne, Lausanne, Switzerland; 19 Department of Psychiatry, Psychotherapy and Psychosomatics, Psychiatry University Hospital Zürich, Zurich, Switzerland; 20 1st Department of Neurology, AHEPA University Hospital, Medical School, Faculty of Health Sciences, Aristotle University of Thessaloniki, Makedonia, Thessaloniki, Greece; 21 Department of Neurobiology, Caring Sciences and Society (NVS), Division of Clinical Geriatrics, Karolinska Institutet, Stockholm, Sweden; 22 Department of Psychiatry in Region Örebro County and School of Medical Sciences, Faculty of Medicine and Health, Örebro University, Örebro, Sweden; 23 Department of Old Age Psychiatry, Psychology & Neuroscience, King’s College, London, UK; 24 University of Oxford, Oxford, UK; 25 Present Address: Johnson and Johnson Medical Ltd., Wokingham, UK; 26 H. Lundbeck A/S, Valby, Denmark; 27 Lübeck Interdisciplinary Platform for Genome Analytics, University of Lübeck, Lübeck, Germany; 28 Paris Brain Institute, ICM, Pitié-Salpêtrière Hospital, Sorbonne University, Paris, France; 29 Neurodegenerative Disorder Research Center, Division of Life Sciences and Medicine, and Department of Neurology, Institute on Aging and Brain Disorders, University of Science and Technology of China and First Affiliated Hospital of USTC, Hefei, People’s Republic of China; 30 Department of Neurodegenerative Disease, UCL Institute of Neurology, London, UK; 31 UK Dementia Research Institute at UCL, London, UK; 32 Hong Kong Center for Neurodegenerative Diseases, Clear Water Bay, Hong Kong, China; 33 Wisconsin Alzheimer’s Disease Research Center, University of Wisconsin School of Medicine and Public Health, University of Wisconsin-Madison, Madison, WI 53792 USA; 34 Department of Neurobiology, Care Sciences and Society, Division of Neurogeriatrics, Karolinska Institutet, Stockholm, Sweden

**Keywords:** Alzheimer’s disease, Choroid plexus, Cerebrospinal fluid, Proteomics, APP knock-in mice, Amyloid-β (Aβ)

## Abstract

**Background:**

Structural and functional changes of the choroid plexus (ChP) have been reported in Alzheimer’s disease (AD). Nonetheless, the role of the ChP in the pathogenesis of AD remains largely unknown. We aim to unravel the relation between ChP functioning and core AD pathogenesis using a unique proteomic approach in mice and humans.

**Methods:**

We used an APP knock-in mouse model, APP^NL-G-F^, exhibiting amyloid pathology, to study the association between AD brain pathology and protein changes in mouse ChP tissue and CSF using liquid chromatography mass spectrometry. Mouse proteomes were investigated at the age of 7 weeks (n = 5) and 40 weeks (n = 5). Results were compared with previously published human AD CSF proteomic data (n = 496) to identify key proteins and pathways associated with ChP changes in AD.

**Results:**

ChP tissue proteome was dysregulated in APP^NL-G-F^ mice relative to wild-type mice at both 7 and 40 weeks. At both ages, ChP tissue proteomic changes were associated with epithelial cells, mitochondria, protein modification, extracellular matrix and lipids. Nonetheless, some ChP tissue proteomic changes were different across the disease trajectory; pathways related to lysosomal function, endocytosis, protein formation, actin and complement were uniquely dysregulated at 7 weeks, while pathways associated with nervous system, immune system, protein degradation and vascular system were uniquely dysregulated at 40 weeks. CSF proteomics in both mice and humans showed similar ChP-related dysregulated pathways.

**Conclusions:**

Together, our findings support the hypothesis of ChP dysfunction in AD. These ChP changes were related to amyloid pathology. Therefore, the ChP could become a novel promising therapeutic target for AD.

**Supplementary Information:**

The online version contains supplementary material available at 10.1186/s12987-024-00555-3.

## Background

Alzheimer’s disease (AD) is characterized by the accumulation of amyloid-beta (Aβ) plaques, followed by the accumulation of neurofibrillary tangles [1–3]. Increasing evidence suggests choroid plexus (ChP) dysfunction in AD [4, 5]. The ChP is a highly vascularized structure, located inside all four brain ventricles, and composed of a monolayer of tight-junction-bound epithelial cells on a basement membrane [6–8], which expresses amyloid precursor protein (APP). The ChP is involved in the production of CSF, transport of ions, proteins, lipids, nutrients and metabolic precursors across the epithelium to the CSF, and clearance of proteins such as Aβ, toxic substances, and metabolites from the CSF. It is also a gateway for immune cell entry into the brain [4, 6–12]. However, the involvement of the ChP in AD pathophysiology remains largely unclear.

Morphological and functional changes of the ChP have been reported in both AD patients and mouse models [4, 5]. Morphological changes in AD include flattening and atrophy of epithelial cells and thickening of the basement membrane and the vessel wall [5, 13–16]. Decreased CSF production and turnover by the ChP have also been reported in AD patients [4, 17], which might lead to impaired CSF Aβ clearance [18–20]. Dysregulation of protein synthesis by the ChP is also observed in AD patients, such as increased production of Aβ [4, 21, 22] and decreased production of transthyretin (TTR) [14], which is protective against cortical Aβ toxicity [23]. Several ChP transcriptomic and proteomic studies in AD patients have been performed, which have indicated dysregulated CSF production and barrier integrity [24], alongside changes in metabolic, immune, and lipids-related pathways [25, 26]. CSF proteomic analysis in AD patients has shown post-mortem abnormal inflammatory signals and protein accumulations, associated with significant remodeling of the ChP [27]. A recent in vivo CSF proteomic study identified a subgroup of persons with AD showing mainly ChP dysfunction [28].

Animal models of AD are critical to understanding disease pathogenesis and pathophysiology, and can offer insights into early stages of disease. Several years ago, new AD knock-in (KI) mouse models were generated including the APP^NL-G-F^ model [29, 30]. These AD KI models offer a new opportunity to study AD pathology in vivo as they closely represent the physiological accumulation of Aβ, without the potential risk of artificial phenotypes associated with the transgenic overexpression of the Aβ precursor protein (APP) present in the first-generation AD models [31]. This APP^NL-G-F^ mouse model presents early and severe Aβ pathology, but does not manifest neurofibrillary tangles or neurodegeneration [32], which makes it an excellent model to study the earliest stages of AD. Moreover, proteomics allows the identification and quantification of proteins in tissues or biological fluids and is a core technique to study the pathophysiological mechanisms underlying a disease [33, 34]. Currently, there are no reports available investigating the ChP tissue proteomic profile in an AD mouse model, while this would be relevant for understanding the mechanisms underlying ChP changes in relation to amyloid pathology in early stages of AD.

The primary aim of the current study was to investigate the ChP changes in relation to AD pathogenesis using ChP tissue proteomics in the APP^NL-G-F^ mouse model. Our secondary aim was to examine how proteomic changes in the mouse ChP were mirrored in the CSF and to compare this to human CSF proteomics findings in AD participants with amyloid but without tau pathology (A+T−) or with amyloid and tau pathology (A+T+) across the clinical spectrum.

## Methods

### Mice

Female APP^NL-G-F^ mice (n = 10), a KI mouse model carrying Arctic, Swedish, and Beyreuther/Iberian mutations [29], and female C57BL/6J mice (wild-type (WT) control; n = 10) were bred in the animal house of the VIB-UGent Center for Inflammation Research and were maintained in ventilated cages, under specific pathogen-free conditions, with ad libitum access to food and water, and a 14-h light/10-h dark cycle. APP^NL-G-F^ and WT mice were sacrificed at 7 or 40 weeks old. The 7 weeks old APP^NL-G-F^ mice represent an early stage of AD; amyloid plaques, microgliosis and astrocytosis start to develop [29]. The 40 weeks old APP^NL-G-F^ mice represent a more advanced stage of AD with amyloid plaques, synaptic loss, microgliosis and astrocytosis [29]. Animal studies were conducted in compliance with governmental and EU guidelines and were approved by the ethical committee of the Faculty of Sciences, Ghent University, Belgium.

AD pathology in our mouse model was confirmed by immunohistochemistry and 3D image analysis (Additional file 1—Results and Additional Fig. 1A–E; Protocols in Additional file 1—Methods and materials) [29, 31, 35].

### Mice CSF and tissue sample isolation

CSF was collected just before sacrifice via the cisterna magna puncture method as described previously [15, 16, 36] and in the Additional file 1—Methods.

To isolate the ChP tissue, mice were transcardially perfused with D-PBS/heparin [0.2% heparin (5.000 IU/ml, Wockhardt)]. Next, both lateral and fourth ventricular ChPs were isolated, snap-frozen in liquid nitrogen and stored at -80 °C until further use [37].

### Mass spectrometry

For proteomic analysis, 5 µl of CSF per mouse and pooled lateral and fourth ventricular ChPs were processed using the PreOmics iST Sample preparation kit (PreOmics Gmbh, Germany), as described by the manufacturer. Peptides were re-dissolved in 20 µl loading solvent A [0.1% trifluoroacetic acid in water/acetonitrile (ACN) (98:2, v/v)] of which 2 µl was injected for LC–MS/MS analysis on an Ultimate 3000 RSLCnano system in-line connected to a Q Exactive HF mass spectrometer (Thermo). More details on the mouse proteomic method can be found in the Additional file 1—Methods.

For the ChP tissue and CSF proteomic analysis respectively, 8519 and 1358 proteins were identified. For further analysis, only the proteins that had at least 3 observations per group [38] were included resulting in 7696 proteins for the ChP tissue proteomics and 319 proteins for the CSF proteomic analyses.

### Classification of ChP protein expression

We labelled the significantly dysregulated proteins in the mouse ChP tissue and CSF proteomic comparisons as being highly expressed in the ChP using published transcriptomic data providing expression levels of genes transcribed in ChP from adult normal mice under physiological conditions [39]. We defined gene expression levels above the 90th percentile as high expression [40].

### Pathway enrichment analysis

Pathway enrichment analyses were performed separately for the decreased and increased significant proteins. QIAGEN Ingenuity Pathway Analysis (IPA) software (QIAGEN Inc., https://digitalinsights.qiagen.com/IPA) [41] was used to find the canonical pathways associated with the significant proteins. Gene Ontology (GO) enrichment analysis was performed using PANTHER (Protein ANalysis THrough Evolutionary Relationships, version 15.0, Los Angeles, CA, USA) [42] in order to identify the biological processes, cellular components and molecular functions related to the significant proteins. The GO enrichment results were validated using ClueGO, a Cytoscape plug-in [43]. All tools use Fisher’s exact test with false discovery rate (FDR; Benjamini–Hochberg procedure [34, 44]) and report only pathways with a FDR corrected p-value < 0.05. To reduce redundancy and facilitate interpretation, we clustered related canonical and GO pathways in broader categories. Further investigation on the functions of specific proteins were also performed using Uniprot [45] and the Human Protein Atlas (proteinatlas.org) [46].

### Human CSF proteomics

To compare mouse findings to human CSF protein changes, we examined data from 496 participants (mean age 68.0 (SD 8.4) years, 54% women) from the European Medical Information Framework for Alzheimer’s Disease Multimodal Biomarker Discovery study (EMIF-AD MBD, n = 346 from 7 cohorts) [47], the Washington University Knight Alzheimer Disease Research Center (ADRC, n = 98) study [48] and the Maastricht BioBank Alzheimer Center Limburg cohort (BB-ACL, n = 52) memory clinic study [49]. We included individuals with availability of CSF Aβ42 (A) and phosphorylated tau (p-tau, T) data, and centrally analysed CSF proteomics (3102 proteins identified; tandem mass tag (TMT) technique). Methods are described previously [34, 50, 51] and provided in Additional File 1. Participants were classified as controls if they had normal cognition (NC) with normal A and T (n = 141). We included individuals across the clinical spectrum with AD pathology, defined as abnormal CSF Aβ1-42 (A+), with either abnormal p-tau (T+) or normal p-tau (T−), resulting in the following groups: NC A+T− [n = 65], mild cognitive impairment (MCI) A+T− [n = 40], Dementia A+T− [n = 17], NC A+T+ [n = 55], MCI A+T+ [n = 114], Dementia A+T+ [n = 64] (more details on participant classification are provided in the Additional File 1—Methods). We tested whether the significant proteins in the human proteomic comparisons were enriched for expression in the ChP using the online database Allen Brain Atlas [52] through Harmonizome [53]. Additionally, we performed expression enrichment analysis using the R package ABAEnrichment [34, 54].

### Statistical analysis

For the mouse study, ChP tissue and CSF protein levels were normalized according to the mean and standard deviation of the respective WT group and compared between groups using ANOVA.

For the human study, CSF protein levels were normalized according to the mean and standard deviation of the control group and compared between groups using ANCOVA corrected for age and sex. In addition, we used linear regression to study associations between human CSF Aβ42 levels (predictors) and CSF levels of proteins associated with the ChP (outcome measures). To this end, Z-scores of local CSF Aβ42 levels were calculated for each centre.

Statistical analyses were performed using R 3.6.2, GraphPad Prism 8.0 and IBM SPSS Statistics version 26.

## Results

### ***Choroid plexus tissue proteomic profile of the APP***^***NL-G-F***^*** mouse model***

To investigate how the ChP changes in relation to AD pathogenesis, we conducted ChP tissue proteomic analysis in the APP^NL-G-F^ mouse model at two distinct ages, i.e., 7 weeks and 40 weeks old.

**Fig. 1:**
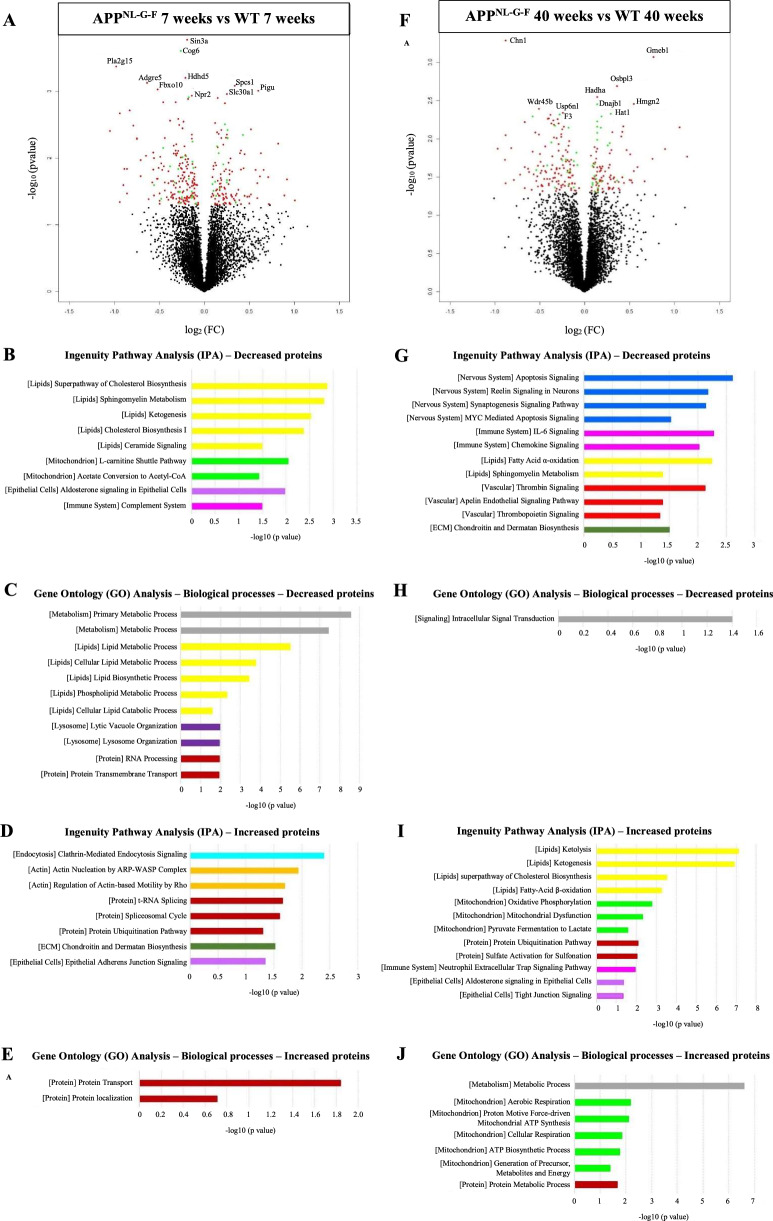
Choroid plexus (ChP) proteomics in APP^NL-G-F^ versus wild-type (WT) mice. **A** Volcano plot displaying the log2 fold-change against the -log10 statistical P-value for the 7 weeks old App^NL-G-F^ compared to their respective WT. Significantly different proteins are red. Significantly different proteins highly expressed by the ChP are green. The top 10 proteins are named. **B** Selected canonical pathways from Ingenuity pathway analysis (IPA) for the decreased proteins in the 7 weeks old APP^NL-G-F^ compared to their respective WT. **C** Selected Gene Ontology (GO) terms including biological process for the decreased proteins in the 7 weeks old APP^NL-G-F^ compared to their respective WT. **D** Selected canonical pathways from IPA for the increased proteins in the 7 weeks old APP^NL-G-F^ compared to their respective WT. **E** Selected GO terms including biological process for the increased proteins in the 7 weeks old APP^NL-G-F^ compared to their respective WT. **F** Volcano plot displaying the log2 fold-change against the −log10 statistical P-value for the 40 weeks old APP^NL-G-F^ compared to their respective WT. Significantly different proteins are red. Significantly different proteins highly expressed by the ChP are green. The top 10 proteins are named. **G** Selected canonical pathways from Ingenuity pathway analysis (IPA) for the decreased proteins in the 40 weeks old APP^NL-G-F^ compared to their respective WT. **H** Selected Gene Ontology (GO) terms including biological process for the decreased proteins in the 40 weeks old APP^NL-G-F^ compared to their respective WT. **I** Selected canonical pathways from IPA for the increased proteins in the 40 weeks old APP^NL-G-F^ compared to their respective WT. **J** Selected GO terms including biological process for the increased proteins in the 40 weeks old APP^NL-G-F^ compared to their respective WT. Pathways linked with lipids are yellow, pathways related to mitochondria and energy metabolism are light green, epithelial cells-linked pathways are light purple, immune system-related pathways are pink, metabolism/signaling-linked pathways are grey, lysosome-related pathways are dark purple, protein-linked pathways are brown, pathways linked with nervous system are blue, vascular-related pathways are red, ECM-related pathways are dark green, endocytosis-related pathways are turquoise and actin-related pathways are orange

ChP tissue proteome analysis of 7 weeks old APP^NL-G-F^ mice showed 184 decreased proteins and 119 increased in the ChP compared to the 7 weeks old WT mice (Fig. 1A, Additional Table 2). The decreased proteins were associated with pathways linked with lipids, mitochondria and the energy metabolism, epithelial cells, immune system (complement), metabolism, lysosomes, and protein transport (Fig. 1B, C). Of the 184 decreased proteins, 25 proteins had a high expression in the ChP based on published transcriptomic data [39] (Fig. 1A). The increased proteins were related to pathways associated with endocytosis, actin, protein formation and modification, extracellular matrix (ECM), and epithelial cells (Fig. 1D, E). Of the 119 increased proteins, 14 proteins had a high expression in the ChP (Fig. 1A). The top 10 proteins with the lowest p-values and their main functions can be found in Table 1.

**Table 1:** Top 10 proteins with the lowest p-values in the comparison of the 7 weeks old APP^NL-G-F^ versus their relative wild-type (WT)

Protein name	APP^NL-G-F^ vs WT (7 weeks)	Highly expressed by the ChP	Main functions
Sin3a	↘		Transcriptional repressor; required for the transcriptional repression of circadian target genes; regulate cell cycle progression; required for cortical neuron differentiation and callosal axon elongation
Cog6	↘	✓	Subunit of the conserved oligomeric Golgi complex; Required for maintaining normal structure and activity of the Golgi apparatus; Involved in protein transport
Pla2g15	↘		Lysophospholipases; Enzyme that act on biological membranes to regulate the multifunctional lysophospholipids
Hdhd5	↘		Predicted to be involved in glycerophospholipid biosynthetic process; Predicted to be active in mitochondria
Adgre5	↘		Member of the EGF-TM7 subfamily of adhesion G protein-coupled receptors; Mediates cell–cell interactions; Plays a role in cell adhesion, in leukocyte recruitment, activation and migration, and in the binding to chondroitin sulfate and the cell surface complement regulatory protein CD55
Spcs1	↗		Component of the signal peptidase complex (SPC); Catalyzes the cleavage of N-terminal signal sequences from nascent proteins as they are translocated into the lumen of the endoplasmic reticulum; Predicted to enable peptidase activity and ribosome binding activity
Fbxo10	↘		Substrate-recognition component of the SCF (SKP1-CUL1-F-box protein)-type E3 ubiquitin ligase complex.; Plays a role in apoptosis, ubiquitination and subsequent lysosomal degradation
Pigu	↗		Fifth subunit of GPI transamidase complex that attaches GPI-anchors to proteins
Npr2	↘		Receptor for natriuretic peptide; Has guanylyl cyclase activity; May play a role in the regulation of skeletal growth
Slc30a1	↗		Zinc ion:proton antiporter; Mediating zinc efflux from cells against its electrochemical gradient protecting them from intracellular zinc accumulation and toxicity

The main functions of the proteins are explained

*Adgre5* Adhesion G protein-coupled receptor E5, *Cog6* Component of oligomeric golgi complex 6, *ChP* choroid plexus, *Fbxo10* F-box protein 10, *Hdhd5* Haloacid dehalogenase like hydrolase domain containing 5, *Npr2* Natriuretic peptide receptor 2, *Pigu* Phosphatidylinositol glycan anchor biosynthesis class U, *Pla2g15* Phospholipase A2 group XV, *Sin3a* SIN3 transcription regulator family member A, *Slc30a1* Solute carrier family 30 member 1, *Spcs1* Signal peptidase complex subunit 1

ChP tissue proteome analysis of 40 weeks old APP^NL-G-F^ mice showed 130 decreased and 107 increased proteins compared to their respective controls (40 weeks old WT mice; Fig. 1F, Additional Table 2). The decreased proteins were associated with pathways linked with nervous system, immune system (interleukins and chemokines), lipids, vascular system and endothelial cells, ECM, as well as signalling (Fig. 1G, H). Of the 130 decreased proteins, 17 proteins had a high expression in the ChP (Fig. 1F). The increased proteins were related to pathways associated with lipids, mitochondria and the energy metabolism, protein modification and degradation, immune system (neutrophils), epithelial cells, and metabolism (Fig. 1I, J). Twenty-two increased proteins were highly expressed in the ChP (Fig. 1F). The top 10 proteins with the lowest p-values and their main functions can be found in Table 2.

**Table 2:** Top 10 proteins with the lowest p-values in the comparison of the 40 weeks old APP^NL-G-F^ versus their relative wild-type (WT)

Protein name	APP^NL-G-F^ vs WT (7 weeks)	Highly expressed by the ChP	Main functions
Chn1	**↘**		GTPase-activating protein for p21-rac and a phorbol ester receptor; Predominantly expressed in neurons; Plays an important role in neuronal signal-transduction mechanisms
Gmeb1	**↗**		Trans-acting factor; Increases sensitivity to low concentrations of glucocorticoids
Osbpl3	**↗**		Intracellular lipid receptors; Associated with both cell and endoplasmic reticulum membranes; May regulate ER morphology; Has a role in regulation of the actin cytoskeleton, cell polarity and cell adhesion
Hadha	**↗**		Alpha subunit of the mitochondrial trifunctional protein; Catalyzes the last three steps of mitochondrial beta-oxidation of long chain fatty acids (major energy-producing process)
Hmgn2	**↗**		Binds nucleosomal DNA and is associated with transcriptionally active chromatin; May help maintain an open chromatin configuration around transcribable genes
Dnajb1	**↗**	✓	Member of the heat shock protein family. Involved in a wide range of cellular events, such as protein folding and oligomeric protein complex assembly; Promote protein folding and prevent misfolded protein aggregation
Wdr45b	**↘**		Component of the autophagy machinery; Controls the major intracellular degradation process by which cytoplasmic materials are packaged into autophagosomes and delivered to lysosomes for degradation
Usp6nl	**↘**		Enables GTPase activator activity and small GTPase binding activity; Involved in several processes, including plasma membrane to endosome transport, positive regulation of GTPase activity and retrograde transport, plasma membrane to Golgi
Hat1	**↗**	✓	Type B histone acetyltransferase; Involved in the rapid acetylation of newly synthesized cytoplasmic histones; Histone acetylation, particularly of histone H4, plays an important role in replication-dependent chromatin assembly
F3	**↘**	✓	Coagulation factor III; Enables cells to initiate the blood coagulation cascades; Platelets and monocytes have been shown to express this coagulation factor under procoagulatory and proinflammatory stimuli

The main functions of the proteins are explained

*Chn1* Chimerin 1, *ChP* choroid plexus, *Dnajb1* DnaJ heat shock protein family (Hsp40) member B1, *F3* Coagulation factor III, *Gmeb1* Glucocorticoid modulatory element binding protein 1, *Hadha* Hydroxyacyl-CoA dehydrogenase trifunctional multienzyme complex subunit alpha, *Hat1* Histone acetyltransferase 1, *Hmgn2* High mobility group nucleosomal binding domain 2, *Osbpl3* Oxysterol binding protein like 3, *Usp6nl* USP6 N-terminal like, *Wdr45b* WD repeat domain 45B

Next, we identified age-dependent proteomic changes at the ChP by comparing the proteomic results of the 7 weeks old APP^NL-G-F^ mice to the ones of the 40 weeks old APP^NL-G-F^ mice. Pathways linked with epithelial cells, mitochondria, protein modification, ECM and lipids were dysregulated at both ages (Fig. 1B–E, G–J). However, only ~ 5% of the dysregulated proteins overlapped in both 7 and 40 weeks old comparisons (Additional Table 2). More specifically, pathways associated with lysosomes, endocytosis, protein formation, actin and complement were uniquely dysregulated in the 7 weeks old APP^NL-G-F^ mice, while pathways associated with the nervous system, immune system (neutrophils, interleukins, chemokines), protein degradation and vascular system were uniquely dysregulated in the 40 weeks old APP^NL-G-F^ mice.

### ***Comparison of ChP tissue with CSF proteomic profiles of the APP***^***NL-G-F***^*** mouse model***

**Fig. 2:**
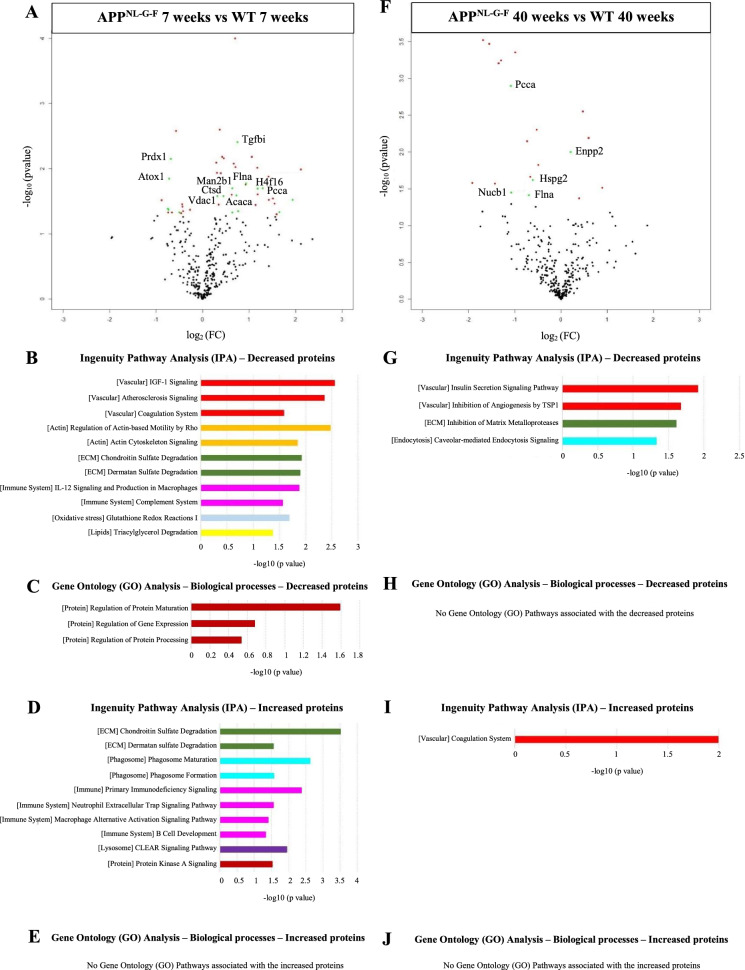
Cerebrospinal fluid (CSF) proteomic profiles of in APP^NL-G-F^ versus wild-type (WT) mice. **A** Volcano plot displaying the log2 fold-change against the −log10 statistical P-value for the 7 weeks old APP^NL-G-F^ compared to their respective WT. Significantly different proteins are red. Significantly different proteins highly expressed by the ChP are green. The top 10 proteins are named. **B** Selected canonical pathways from Ingenuity pathway analysis (IPA) for the decreased proteins in the 7 weeks old APP^NL-G-F^ compared to their respective WT. **C** Selected Gene Ontology (GO) terms including biological process for the decreased proteins in the 7 weeks old APP^NL-G-F^ compared to their respective WT. **D** Selected canonical pathways from IPA for the increased proteins in the 7 weeks old APP^NL-G-F^ compared to their respective WT. **E** Selected GO terms including biological process for the increased proteins in the 7 weeks old APP^NL-G-F^ compared to their respective WT. **F** Volcano plot displaying the log2 fold-change against the −log10 statistical P-value for the 40 weeks old APP^NL-G-F^ compared to their respective WT. Significantly different proteins are red. Significantly different proteins highly expressed by the ChP are green. The top 10 proteins are named. **G** Selected canonical pathways from Ingenuity pathway analysis (IPA) for the decreased proteins in the 40 weeks old APP^NL-G-F^ compared to their respective WT. **H** Selected Gene Ontology (GO) terms including biological process for the decreased proteins in the 40 weeks old APP^NL-G-F^ compared to their respective WT. **I** Selected canonical pathways from IPA for the increased proteins in the 40 weeks old APP^NL-G-F^ compared to their respective WT. **J** Selected GO terms including biological process for the increased proteins in the 40 weeks old APP^NL-G-F^ compared to their respective WT. Vascular-related pathways are red, actin-related pathways are orange, ECM-related pathways are dark green, immune system-related pathways are pink, pathways associated with oxidative stress are light blue, pathways linked with lipids are yellow, protein-linked pathways are brown, endocytosis/phagocytosis-related pathways are turquoise and lysosome-related pathways are dark purple

Next, we performed CSF proteomic analysis to test whether pathological changes at the ChP are mirrored in the CSF of the APP^NL−G−F^ mouse model (Fig. 2). Results of the mouse CSF proteomic analysis are described in Additional file 1—Results, Fig. 2 and Additional Table 3.

We investigated the overlap between ChP tissue and CSF dysregulated proteins for the 7 and 40 weeks old mice comparisons with controls. Two hundred eighty-nine proteins were identified in both ChP tissue and CSF (8% showed correlation between ChP and CSF). However, there was no overlap in dysregulated proteins in ChP tissue and CSF at both ages. When we compared processes associated with the dysregulated proteins in ChP tissue and CSF, the 7 weeks old APP^NL-G-F^ mouse showed overlap in dysregulated pathways associated with ECM, lysosomes, protein processing, actin, lipids and complement, while the 40 weeks old APP^NL-G-F^ mouse showed only overlap in dysregulated pathways linked to ECM and the vascular system.

### Comparison of mouse ChP and CSF proteomic results with human CSF proteomics

**Fig. 3:**
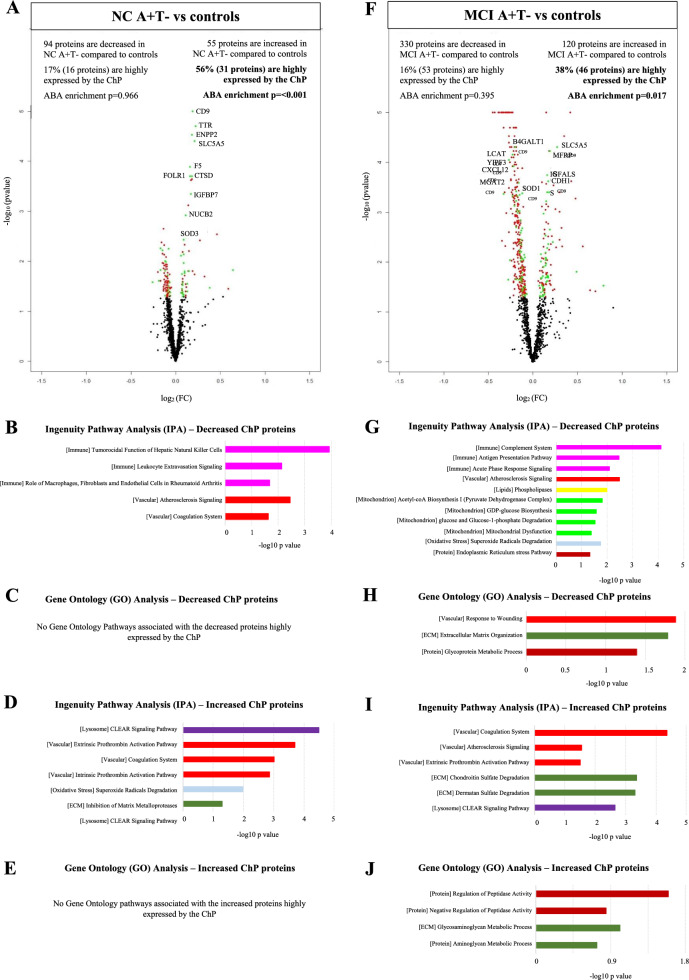
Cerebrospinal fluid (CSF) proteomic profiles and associated ChP pathways in A+T− individuals with normal cognition (NC) and mild cognitive impairment (MCI). (A) Volcano plot displaying the log2 fold-change against the −log10 statistical P-value for the comparison NC A+T− vs controls. Significantly different proteins are red. Significantly different proteins highly expressed by the ChP are green. The top 10 proteins highly expressed by the ChP are named. The number of proteins highly expressed by the ChP, as well as the gene expression enrichment in the ChP (ABAenrichment) p-value, are displayed. **B** Selected canonical pathways from Ingenuity pathway analysis (IPA) for the decreased proteins highly expressed by the ChP in the comparison NC A+T− vs controls. **C** Selected Gene Ontology (GO) terms including biological process for the decreased proteins highly expressed by the ChP in the comparison NC A+T− vs controls. **D** Selected canonical pathways from IPA for the increased proteins highly expressed by the ChP in the comparison NC A+T− vs controls. **E** Selected GO terms including biological process for the increased proteins highly expressed by the ChP in the comparison NC A+T− vs controls. **F** Volcano plot displaying the log2 fold-change against the −log10 statistical P-value for the comparison MCI A+T− vs controls. Significantly different proteins are red. Significantly different proteins highly expressed by the ChP are green. The top 10 proteins highly expressed by the ChP are named. The number of proteins highly expressed by the ChP, as well as the gene expression enrichment in the ChP (ABAenrichment) p-value, are displayed. **G**- Selected canonical pathways from Ingenuity pathway analysis (IPA) for the decreased proteins highly expressed by the ChP in the comparison MCI A+T− vs controls. **H** Selected Gene Ontology (GO) terms including biological process for the decreased proteins highly expressed by the ChP the comparison MCI A+T− vs controls. **I** Selected canonical pathways from IPA for the increased proteins highly expressed by the ChP in the comparison MCI A+T− vs controls. **J** Selected GO terms including biological process for the increased proteins highly expressed by the ChP in the comparison MCI A+T− vs controls. Immune-related pathways are pink, vascular-related pathways are red, pathways associated with lysosomes are dark purple, pathways associated with oxidative stress are light blue, pathways related to ECM are dark green, pathways linked with lipids are yellow, pathways related to energy metabolism and mitochondria are light green and protein-linked pathways are brown

To further understand how ChP-related changes in the proteomes of the APP^NL-G-F^ mouse model reflect those observed in human patients, we next compared the mouse proteomic results (both CSF and ChP tissue) to the CSF proteomic results in humans with AD. In A+T− and A+T+ individuals with NC, MCI or AD dementia, we first selected CSF proteins that differed between AD patients and controls. Next, we tested which of the proteins had a high expression in the ChP according to the Human Brain Atlas, to define the proteins involved in ChP functioning (Additional Table 4). Among AD CSF proteins with an increased concentration relative to controls, a significant number of proteins were highly expressed by the ChP in NC A+T− (56%, ABAenrichment p ≤ 0.001, Fig. 3A) and MCI A+T− (38%, ABAenrichment p = 0.017, Fig. 3F), but not in AD dementia (33%, ABAenrichment p = 0.817, Additional Fig. 2A). The decreased proteins were not enriched for expression in the ChP. The ChP-enriched dysregulated proteins in persons with A+T− were different along the clinical spectrum (Additional Fig. 3, Additional Table 4). Nonetheless, in NC and MCI A+T−, the increased proteins highly expressed by the ChP were associated with lysosomes, vascular system, ECM, oxidative stress and protein processing or degradation (Fig. 3D-E and I-J). In individuals with A+T+, we did not find significant enrichment for expression in the ChP (15 to 35% of significant proteins highly expressed by the ChP, Additional Fig. 4A–C). Further analysis therefore focused on the A+T− groups. More details on the results of the human CSF proteomics analysis can be found in Additional file 1—Results.

**Fig. 4:**
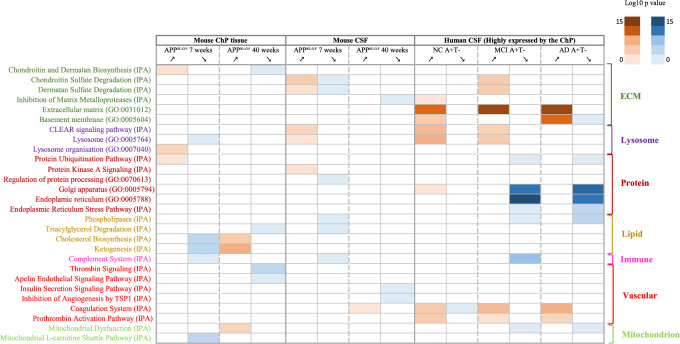
Selected Ingenuity pathway analysis (IPA) canonical pathways or Gene Ontology (GO) biological/cellular processes enriched for proteins in the different comparisons of the paper with decreased (blue) or increased (red) concentrations relative to controls. The comparisons include the choroid plexus (ChP) proteomic analysis of 7 weeks old APP^NL-G-F^ mice versus\wild-type (WT), the ChP proteomic analysis of the 40 weeks old APP^NL-G-F^ mice versus their relative WT, the cerebrospinal fluid (CSF) proteomic analysis of the 7 weeks APP^NL-G-F^ mice versus their relative WT, the CSF proteomic analysis of the 40 weeks old APP^NL-G-F^ mice versus their relative WT, the CSF proteomic analysis of human with normal cognition (NC) and abnormal amyloid-β 42 (A) levels and normal phosphorylated tau (T) levels (A+T−) versus controls, the CSF proteomic analysis of individuals with mild cognitive impairment (MCI) A+T− versus controls and the CSF proteomic analysis of Alzheimer’s dementia (AD) A+T− versus controls. P-values are presented and scaled based on the scale in the right of the graphs. ECM-related pathways are dark green, lysosome-related pathways are dark purple, protein-linked pathways are brown, pathways linked with lipids are yellow, immune system-related pathways are pink, vascular-related pathways are red and pathways related to mitochondria and energy metabolism are light green

#### ChP changes in AD in both mouse (ChP tissue and CSF) and human (CSF) proteomes

Figure 4 presents an overview of the overlap in dysregulated ChP-associated pathways identified in AD mouse CSF, mouse ChP tissue and human CSF proteomes. These analyses showed ChP involvement in AD with protein changes related to the ECM, lysosomes, protein processing, lipids, complement, vascular system and mitochondria.

**Fig. 5:**
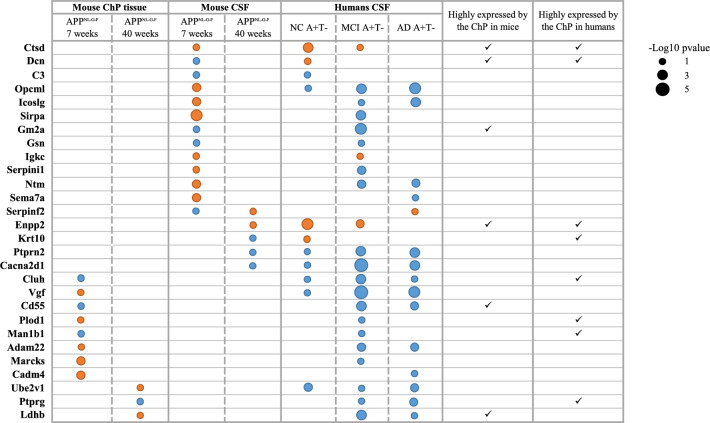
Choroid plexus (ChP)-related proteins dysregulated in both mice (ChP tissue or CSF) and humans (CSF). The comparisons include the ChP tissue proteomic analysis of the 7 weeks old APP^NL-G-F^ mice versus their relative wild-type (WT), the ChP proteomic analysis of the 40 weeks old APP^NL-G-F^ mice versus their relative WT, the cerebrospinal fluid (CSF) proteomic analysis of the 7 weeks old APP^NL-G-F^ mice versus their relative WT, the CSF proteomic analysis of the 40 weeks old APP^NL-G-F^ mice versus their relative WT, the CSF proteomic analysis of human with normal cognition (NC) and abnormal amyloid-β 42 (A) levels and normal phosphorylated tau (T) levels (A+T−) versus controls, the CSF proteomic analysis of individuals with mild cognitive impairment (MCI) A+T− versus controls and the CSF proteomic analysis of Alzheimer’s dementia (AD) A+T− versus controls. P-values are presented and scaled based on the dot scale in the right of the graphs. A blue dot means decreased concentrations relative to controls and a red dot means increased concentrations relative to controls. *AD* Alzheimer’s dementia, *Adam22* ADAM metallopeptidase domain 22, *C3* Complement C3, *Cacna2d1* Calcium voltage-gated channel auxiliary subunit alpha2delta 1, *Cadm4* Cell adhesion molecule 4, *Cluh* clustered mitochondria protein homolog, *CSF* cerebrospinal fluid, *ChP* choroid plexus, *Ctsd* cathepsin D, *Dcn* decorin, *Enpp2* Autotaxin, *Gm2a* Ganglioside GM2 activator, *Gsn* Gelsolin, *Icoslg* Inducible T cell costimulator ligand, *Igkc* Immunoglobulin kappa constant, *Krt10* Keratin 10, *Ldhb* Lactate dehydrogenase B, *Man1b1* Endoplasmic reticulum mannosyl-oligosaccharide 1,2-alpha-mannosidase protein, *Marcks* Myristoylated alanine rich protein kinase C substrate, *MCI* Mild cognitive impairment, *NC* Normal cognition, *Ntm* Neurotrimin, *Opcml* Opioid binding protein/cell adhesion molecule like, *Plod1* procollagen-lysine,2-oxoglutarate 5-dioxygenase 1 protein, *Ptprg* Receptor-type tyrosine-protein phosphatase gamma, *Ptprn2* Protein tyrosine phosphatase receptor type N2, *Sema7a* Semaphorin 7A, *Serpinf2* Serpin family F member 2, *Serpini1* Serpin family I member 1, *Sirpa* Signal regulatory protein alpha, *Ube2v1* Ubiquitin conjugating enzyme E2 V1, *Vgf* Vgf nerve growth factor inducible

To investigate the similarity of CSF protein changes associated with ChP functioning in AD across species, we compared human and mouse CSF proteomics. There were 215 proteins commonly identified in both datasets. Seventeen CSF proteins were dysregulated in both mice and humans and relevant for ChP functioning (Fig. 5; 5 proteins decreased, 3 proteins increased, and 9 proteins in opposite direction), which were associated with lysosomes, ECM, immune system (complement, T cells, B cells, immunoglobulins, cytokines), cell adhesion, lipids, actin and microtubule and hemostasis (Table 3). Out of those 17 proteins, 5 were highly expressed by the ChP (Fig. 5, Table 3).

**Table 3:** Linear regression table between the 28 choroid plexus (ChP)-related proteins and CSF amyloid-β 42 (Aβ42) levels

P-value Beta	CSF Aβ42	Highly expressed by the ChP	Main functions
Adam22	**0.001** **0.129**		Membrane-anchored protein; Implicated in cell–cell and cell–matrix interactions; May function as an integrin ligand; It has no metalloprotease activity
C3	**0.021** **0.093**		Complement component; Plays a central role in the activation of complement system (both classical and alternative)
Cacna2d1	**< 0.001** **0.284**		Subunit of voltage-dependent calcium channels; Mediate the influx of calcium ions into the cell upon membrane polarization
Cadm4	**0.014** **0.099**		Cell–cell adhesion protein; Involved in negative regulation of protein phosphorylation, regulation of Rac protein signal transduction and regulation of wound healing
Cd55	**0.004** **0.116**		Glycoprotein involved in the regulation of the complement cascade; Inhibits complement activation by destabilizing and preventing the formation of C3 and C5 convertases
Cluh	**0.011** **0.157**	✓	mRNA-binding protein; involved in proper cytoplasmic distribution of mitochondria
Ctsd	**< 0.001** − **0.272**	✓	Lysosomal protease; plays a role in amyloid protein precursor (APP) processing; is the principal Aβ-degrading protease
Dcn	**< 0.001** − **0.186**	✓	Extracellular matrix protein; plays a role in collagen fibril assembly
Enpp2	**< 0.001** − **0.323**	✓	Hydrolase; Hydrolyzes lysophospholipids to produce the signaling molecule lysophosphatidic acid (LPA)
Gm2a	**< 0.001** **0.253**	✓	Lipid transport protein; acts as a substrate specific co-factor for the lysosomal enzyme beta-hexosaminidase A; important for the normal lysosomal function
Gsn	0.060 0.076		Actin-modulating protein; Has functions in both assembly and disassembly of actin filaments
Icoslg	**< 0.001** **0.149**		Ligand for the T-cell-specific cell surface receptor ICOS; Acts as a costimulatory signal for T-cell proliferation and cytokine secretion; Induces also B-cell proliferation and differentiation into plasma cells
Igkc	**0.018** − **0.097**		Constant region of immunoglobulin light chains
Krt10	**0.039** − **0.084**	✓	Keratin; forms the intermediate filament, which, along with actin microfilaments and microtubules, compose the cytoskeleton of epithelial cells
Ldhb	**0.009** **0.107**		B subunit of lactate dehydrogenase enzyme; which catalyzes the interconversion of pyruvate and lactate with concomitant interconversion of NADH and NAD + in a post-glycolysis process
Man1b1	0.157 0.066	✓	Glycosidase; found in the ER quality control compartment; involved in glycoprotein quality control targeting of misfolded glycoproteins for degradation; involved in N-glycan biosynthesis
Marcks	**0.042** **0.084**		Substrate for protein kinase C; Actin filament crosslinking protein; Involved in cell motility, phagocytosis, membrane trafficking and mitogenesis
Ntm	**< 0.001** **0.233**		Neural cell adhesion protein
Opcml	**< 0.001** **0.224**		Cell adhesion protein
Plod1	**< 0.001** − **0.199**	✓	Endoplasmic reticulum hydroxylase; catalyzes hydroxylation of lysine residues in collagen alpha chains; is required for normal assembly and cross-linking of collagen fibrils
Ptprg	**< 0.001** **0.180**	✓	Protein phosphatase; signaling molecules that regulate a variety of cellular processes including cell growth, differentiation and mitotic cycle
Ptprn2	**< 0.001** **0.340**		Plays a role in vesicle-mediated secretory processes; Plays a role in insulin secretion in response to glucose stimuli
Sema7a	0.087 0.070		Semaphorin protein; Promotes production of pro-inflammatory cytokines by monocytes and macrophages. Plays an important role in modulating inflammation and T-cell-mediated immune responses
Serpinf2	**0.025** − **0.091**		Protease inhibitor; Major inhibitor of plasmin; Major role in regulating the blood clotting pathway
Serpini1	**< 0.001** **0.143**		Serine proteinase inhibitor; Reacts with and inhibits tissue-type plasminogen activator; Plays a role in the regulation of axonal growth and the development of synaptic plasticity
Sirpa	**< 0.001** **0.216**		Supports adhesion of cerebellar neurons, neurite outgrowth and glial cell attachment; Important during synaptogenesis and in synaptic function; Mediates negative regulation of phagocytosis, mast cell activation and dendritic cell activation
Ube2v1	0.092 0.082		Mediates transcriptional activation of target genes; Plays a role in the control of progress through the cell cycle and differentiation
Vgf	**< 0.001** **0.416**		Plays many roles in neurogenesis and neuroplasticity

The main functions of the proteins are explained

Values represent p-value and regression coefficient Beta. Significant P-values (< 0.05) are bold. All measures were transformed in Z-scores before linear regression

*Aβ42* amyloid beta 42, *Adam22* ADAM metallopeptidase domain 22, *C3* Complement C3, *Cacna2d1* Calcium voltage-gated channel auxiliary subunit alpha2delta 1, *Cadm4* Cell adhesion molecule 4, *Cluh* clustered mitochondria protein homolog, *ChP* choroid plexus, *Ctsd* cathepsin D, *Dcn* decorin, *Enpp2* Autotaxin, *Gm2a* Ganglioside GM2 activator, *Gsn* Gelsolin, *Icoslg* Inducible T cell costimulator ligand, *Igkc* Immunoglobulin kappa constant, *Krt10* Keratin 10, *Ldhb* Lactate dehydrogenase B, *Man1b1* Endoplasmic reticulum mannosyl-oligosaccharide 1,2-alpha-mannosidase protein, *Marcks* Myristoylated alanine rich protein kinase C substrate, *Ntm* Neurotrimin, *Opcml* Opioid binding protein/cell adhesion molecule like, *Plod1* procollagen-lysine,2-oxoglutarate 5-dioxygenase 1 protein, *Ptprg* Receptor-type tyrosine-protein phosphatase gamma, *Ptprn2* Protein tyrosine phosphatase receptor type N2, *Sema7a* Semaphorin 7A, *Serpinf2* Serpin family F member 2, *Serpini1* Serpin family I member 1, *Sirpa* Signal regulatory protein alpha, *Ube2v1* Ubiquitin conjugating enzyme E2 V1, *Vgf* Vgf nerve growth factor inducible

Next, to understand to what extent proteomic changes in AD mice ChP tissue are present in AD human CSF, we compared human CSF proteomics to mice ChP tissue proteomics. This also allowed us to identify relevant ChP related proteins in humans beyond those highly expressed by the ChP. There were 691 proteins commonly identified in both datasets. Eleven proteins were dysregulated in both mice and humans (Fig. 5; 4 proteins decreased and 7 proteins in opposite direction), which were associated with mitochondria and energy metabolism, nervous system, complement, ECM, protein formation, folding and modification, cell–cell and cell–matrix interactions and actin (Table 3). Six proteins were highly expressed by the ChP (Fig. 5, Table 3).

Together, we identified 28 proteins associated with ChP functioning and dysregulated in both AD mouse and human proteomes (17 in mouse versus human CSF proteomes; 11 in mouse ChP tissue versus human CSF proteomes; see above). Next, we investigated the association between the levels of those 28 proteins and CSF Aβ42 in the overall human dataset (Table 3). Globally, reduced levels of 17 proteins were associated with lower, thus more abnormal, Aβ42. Those proteins were associated with the nervous system, energy metabolism, protein formation, folding and modification, lipids, cell–cell adhesion and immune system (complement, T cells). We further observed, for 7 proteins, that increased levels were associated with more abnormal Aβ42 levels. Those proteins were linked to the lysosomes, ECM and collagen, mitochondria, immunoglobulins and cytoskeleton. Four proteins were not associated with Aβ42 levels.

## Discussion

We aimed to investigate the changes of the ChP in relation to the pathogenesis of AD using ChP tissue proteomics in APP^NL-G-F^ mice, and compared this to CSF proteomic profiles in both AD mice and humans. In ChP tissue of mice at both 7 and 40 weeks old, pathways linked with epithelial cells, mitochondria, protein modification, extracellular matrix and lipids were dysregulated, while pathways associated with lysosome, endocytosis, protein formation, actin and complement were mainly seen at 7 weeks, and pathways associated with nervous system, interleukins and neutrophils, protein degradation and vascular system were mainly found at 40 weeks. Similar results were observed in the CSF of APP^NL-G-F^ mice, as well as of human AD patients with amyloid but without tau pathology. Our findings highlight ChP dysfunction in relation to amyloid pathology, which is relevant for AD treatment strategies.

A high number of dysregulated proteins were found in the ChP tissue of the APP^NL-G-F^ AD mouse model, already at early disease stages (7 weeks old). The ChP protein changes were linked to multiple dysregulated pathways of which several showed consistency across ages, while some differed across ages. Findings are consistent with a previous ChP transcriptomic study in another AD mouse model (J20), in which they found a significant number of dysregulated genes already at an early AD stage (3 months), with differences across ages [55]. This suggests a dynamic and complex process underlying ChP dysfunction in AD.

The dysregulated pathways observed in AD and linked with epithelial cells, vascular system, ECM, lysosome, mitochondria and protein processing can be associated with changes in the morphology of the ChP. Flattening and atrophy of ChP epithelial cells, as well as a decline of epithelial tight junctions, in mice and humans with AD have been reported previously, and might be linked with increased Aβ deposits [5, 13, 14, 56]. Changes in the ChP basement membrane, a thin layer of ECM, in AD has also been previously reported, with increased thickness (due to an accumulation of collagen) and irregularity, which reduce the permeability, plasma ultrafiltration, ChP epithelial oxygenation and CSF formation [4, 14, 57]. A high number of vesicles with lysosomal characteristics are present in the ChP cytoplasm [58]. Multiple human and mouse AD studies have reported impairment of autophagy–lysosomal pathway, which is partly responsible for the accumulation of Aβ [59–62]. A high density of mitochondria, Golgi apparatus and a smooth endoplasmic reticulum can be found in the ChP epithelial cells [58]. In AD, Golgi defects and endoplasmic reticulum stress have been reported, leading to a dysfunction of folding, trafficking, processing, and sorting of proteins [63, 64], while a defect in mitochondrial enzyme activity of the ChP epithelial cells can result in decreased transport across the epithelial cells and thus has implications in Aβ clearance in the ChP of AD patients [65, 66]. On the other hand, Aβ itself can also impair mitochondrial function in the ChP [67].

The dysregulated pathways related to lipids and immune system observed in AD can be associated with functional dysfunction of the ChP. The ChP plays a crucial role in the transport of lipids from the blood to the CSF [68] and acts as a reservoir for multiple types of immune cells [10]. Previous studies on AD patients reported the presence of complement components as well as activation of the complement cascade in the ChP [69, 70].

While the protein changes in tissue were similar to those in CSF on a pathway level, at the protein level, ChP tissue changes were not directly reflected in the CSF in our AD mouse model. This could be linked with the ChP epithelial cell and tight junction alterations that we found in our ChP tissue proteomics analysis, which may indicate changes in blood-CSF barrier permeability [56, 71]. Furthermore, a previous mouse study showed that intracerebroventricular injection of Aβ1-42 oligomers rapidly affected ChP epithelial cells and tight junctions, which were associated with an increase in blood-CSF barrier leakage [15]. Alternatively, as CSF has been isolated in sedated mice while tissue has been extracted after death, this could have resulted in differences in changes in proteins in CSF and ChP tissue. Future studies are needed to further explore the AD-related changes in ChP permeability in relation to changes in epithelial cells, epithelial tight junctions and epithelial transport proteins. Similarly, a small overlap was observed at the protein level between mice and humans for significant CSF proteins associated with ChP functioning.

We found a correlation with CSF amyloid levels for most proteins that were associated with ChP changes in both mouse and human. In line with previous publications, this supports a causal relationship between ChP protein changes and amyloid pathology in AD [4, 17–22]. Several studies in both AD patients and AD mouse models have reported Aβ deposits in the ChP epithelial cells and stroma surrounding capillaries in AD [5, 14, 67, 72], which could lead to morphological and functional alterations of ChP [15, 67, 73].

Our study has several strengths and limitations. To the best of our knowledge, this is the first study reporting ChP tissue proteomic analysis in an AD mouse model. We used APP^NL-G-F^ knock-in mice, which is an AD model exhibiting amyloid pathology without the typical APP overexpression artefacts. Another main strength of this study is our translational approach. We compared ChP tissue proteomics in AD mice to CSF proteomics in AD mice and humans to gain novel insights into the role of the ChP in AD pathogenesis. Yet, for comparisons between these findings, this resulted in a smaller set of overlapping proteins that could be studied. This could have led to missing key pathways and proteins associated with the ChP implication in AD. Moreover, we made use of a unique large dataset for the human CSF proteomic analyses which covered the whole clinical spectrum. Yet, for the human dataset, we used ChP expression to define the proteins involved in the functioning of the ChP. While this is the best proxy at hand, this may have resulted in less identified proteins that play a role in the ChP. Future research should validate our findings using post-mortem human ChP samples, from individuals with various extents of AD pathology. Moreover, the exclusive use of female mice may potentially limit the generalizability of our findings to both sexes. Previous publications showed earlier AD pathology onset in female mice compared to male mice, with more profound amyloidosis and a higher percentage of astrocytes in the cortex and hippocampus of 18-month old female APP^NL-G-F^ mice compared to male mice [74]. It could also be that early ChP changes are more pronounced in female mice.

## Conclusions

Together, our findings support the hypothesis of dysregulated ChP functioning in AD. These ChP changes were already present at early stages of AD, were related to amyloid pathology, and were related to similar key pathways across the disease trajectory for mice and the clinical trajectory of humans. Key pathways related to the ChP dysfunction in AD are associated with ECM, lysosomes, lipids, protein processing, complement, vascular system and mitochondria. Our results further contribute towards better pathophysiological characterization of the involvement of the ChP in AD. It has implications for drug development, as ChP changes were already present at early stages of AD and associated with amyloid pathology. Addressing fundamental mechanisms linked to ChP functioning, such as ECM-related pathways, lysosomal pathways, or vascular pathways, may hold therapeutic promise.

### Supplementary Information

**Additional file 1. **This file provides supplemental information on the methods, supplementary results and supplementary figures.

**Additional table 2. **Dysregulated proteins for each comparison in the whole list of identified proteins in mouse ChP tissue.

**Additional table 3. **Dysregulated proteins for each comparison in the whole list of identified proteins in mouse CSF.

**Additional table 4. **Dysregulated proteins for each comparison in the whole list of identified proteins in human CSF.

## Data Availability

The mouse mass spectrometry proteomics data have been deposited to the ProteomeXchange Consortium via the PRIDE partner repository with the dataset identifier PXD052590. The data underlying this article will be shared on reasonable request to the corresponding author. The EMIF-AD MBD mass spectrometry proteomics data have been deposited to the ProteomeXchange Consortium via the PRIDE partner repository with the dataset identifiers PXD019910 and 10.6019/PXD019910.

## Abbreviations

A: Amyloid
Aβ: Amyloid-beta
AD: Alzheimer’s disease
ADRC: Alzheimer Disease Research Center
BB-ACL: BioBank Alzheimer Center Limburg
ChP: Choroid plexus
CSF: Cerebrospinal fluid
ECM: Extracellular matrix
EMIF-AD MBD: European Medical Information Framework for Alzheimer’s Disease Multimodal Biomarker Discovery
FDR: False discovery rate
GO: Gene Ontology
IPA: Ingenuity pathway analysis
KI: Knock-in
MCI: Mild cognitive impairment
NC: Normal cognition
P-tau: Phosphorylated tau
T: Tau
TMT: Tandem mass tag
TTR: Transthyretin
WT: Wild-type
