# Supplementary material for: Involvement of the choroid plexus in Alzheimer’s disease pathophysiology: findings from mouse and human proteomic studies
Source: Fluids Barriers CNS. 2024 Jul 18;21:58. doi: 10.1186/s12987-024-00555-3 (PMC11256635; doi:10.1186/s12987-024-00555-3)
Supplement: Supplementary file 1 — Additional file 1. This file provides supplemental information on the methods, supplementary results and supplementary figures. [file 12987_2024_555_MOESM1_ESM.docx]

**Additional information**

**Involvement of the choroid plexus in Alzheimer’s disease pathophysiology: Findings from mouse and human proteomic studies.**

Aurore Delvenne, Charysse Vandendriessche, Johan Gobom, Marlies Burgelman, Pieter Dujardin, Clint De Nolf, Betty M. Tijms, Charlotte E. Teunissen, Suzanne E. Schindler, Frans Verhey, Inez Ramakers, Pablo Martinez-Lage, Mikel Tainta, Rik Vandenberghe, Jolien Schaeverbeke, Sebastiaan Engelborghs, Ellen De Roeck, Julius Popp, Gwendoline Peyratout, Magda Tsolaki, Yvonne Freund-Levi, Simon Lovestone, Johannes Streffer, Lars Bertram, Kaj Blennow, Henrik Zetterberg, Pieter Jelle Visser, Roosmarijn E. Vandenbroucke and Stephanie J.B. Vos.

**Contents**

Additional Methods

Additional Results

References

Additional Tables and Figures

**Additional Methods**

**CSF sample isolation**

CSF was collected via the cisterna magna puncture method. Briefly, capillaries to isolate CSF were made from borosilicate glass capillary tubes (B100-75-15; Sutter Instruments) using the Sutter P-87 flaming micropipette puller (pressure 330 Pa, heat index 300). Just before CSF isolation, mice were anesthetized with 200 µl of ketamine/xylazine (100 mg/kg ketamine; 20 mg/kg xylazine). An incision was made inferior to the occiput and disinfected with 70% ethanol. The dura mater was exposed by separating the muscle tissue on the dorsal side of the skull. Next, the animal was mounted at an angle of 135° and CSF was collected by puncturing the dura mater of the cisterna magna using the capillary needle.

**Mouse mass spectrometry**

Trapping was performed at 20 μl/min for 2 min in loading solvent A on a 5 mm trapping column (Thermo scientific, 300 μm internal diameter (I.D.), 5 μm beads). The peptides were separated on a 250 mm Aurora Ultimate, 1.7µm C18, 75 µm inner diameter (Ionopticks) kept at a constant temperature of 45°C. Peptides were eluted by a non-linear gradient starting at 1% MS solvent B reaching 33% MS solvent B (0.1% FA in water/acetonitrile (2:8, v/v)) in 100 min, 55% MS solvent B (0.1% FA in water/acetonitrile (2:8, v/v)) in 135 min, 70% MS solvent B in 145 minutes followed by a 5-minute wash at 70% MS solvent B and re-equilibration with MS solvent A (0.1% FA in water). The mass spectrometer was operated in data-independent mode, automatically switching between MS and MS/MS acquisition. Full-scan MS spectra ranging from 375-1500 m/z with a target value of 5E6, a maximum fill time of 50 ms and a resolution at of 60,000 were followed by 30 quadrupole isolations with a precursor isolation width of 10 m/z for HCD fragmentation at an NCE of 30% after filling the trap at a target value of 3E6 for maximum injection time of 45 ms. MS2 spectra were acquired at a resolution of 15,000 at 200 m/z in the Orbitrap analyser without multiplexing. The isolation intervals ranging from 400 – 900 m/z, without overlap were created with the Skyline software tool.

The polydimethylcyclosiloxane background ion at 445.120028 Da was used for internal calibration (lock mass) and QCloud (1) was used to control instrument longitudinal performance during the project.

LC-MS/MS runs of all samples were searched together using the DiaNN algorithm (version 1.8.1), library free. Spectra were searched against the mouse reference protein sequences in the Swiss-Prot database (database release version of 2022_01), containing 21,986 sequences (www.uniprot.org). Enzyme specificity was set as C-terminal to arginine and lysine, also allowing cleavage at proline bonds with a maximum of two missed cleavages. Variable modifications were set to oxidation of methionine residues and acetylation of protein N-termini. Mainly default settings were used, except for the addition of a 400-900 m/z precursor mass range filter, the match between runs (MBR) option and the MS1 and MS2 mass accuracy was set to 10 and 20 ppm respectively.

**Immunohistochemistry and image analysis**

In order to validate amyloidosis in the used APP^NL-G-F^ mouse model, immunostaings against Aβ were performed. Therefore, mice were transcardially perfused with 4% paraformaldehyde, brain samples were isolated and post-fixed in 4% paraformaldehyde overnight at 4 °C. After dehydration, samples were embedded in paraffin and stored at room temperature until further use. The paraffin embedded brain samples were cut into 5 μm sections (HM 340 E, Thermo Scientific). Sections were dewaxed using Varistain (Leica Autostainer XL (Leica, Wetzlar, Germany)), followed by an antigen retrieval step using citrate buffer (Vector; H-3300) and wash steps with PBS. Next, samples were blocked with 5% normal goat serum in PBT (0.3% Triton X-100 in PBS) for an hour at room temperature. Brain sections were stained overnight with primary antibody against amyloid β-peptide (Aβ) (clone 6E10, 803001, BioLegend; 1/500) at 4 °C. Then, slides were washed with PBS and incubated with secondary antibody for 2 hours at room temperature (Alexa Flour 488 goat anti-mouse, A11001, Thermo Fisher Scientific). After washing with PBS, slides were stained with DAPI for 20 minutes at room temperature (4’,6-Diamidino-2-Phenylindole; 62248, Thermo; 1/1000) and mounted with n-PVA-DABCOmounting medium. Image acquisition of fluorescently stained sections was performed using a Zeiss LSM780 confocal microscope (Carl Zeiss, Oberkochen, Germany).

**Astrocytes and microglia three-dimensional reconstruction**

Astrocytes and microglia three-dimensional (3D) reconstructions were performed to evaluate the viability of our APP^NL-G-F^ mouse model. Tissue samples were fixed in paraformaldehyde (PFA) overnight at 4°C. After fixation, the tissue was washed with PBS and embedded in 5% low-gelling agarose (Sigma Aldrich A4018-50G). Vibratome brain sections of 50μm were blocked with 5% normal goat serum in PBT (0.3% Triton X-100 in PBS) for an hour at room temperature and stained overnight with either anti-IBA1 (microglia; 019-19741, Wako Chemicals;1/500) or anti-GFAP (astrocytes; ab53554, Abcam; 1/500) antibodies at 4 °C. After washing steps, the sections were incubated with secondary antibodies for 2 hours at room temperature (Alexa Fluor 633 goat anti-rabbit, A21070, Thermo Fisher Scientific; 1/300). Finally, the slides were washed and mounted with n-propyl mounting medium. Z-stack images were taken with a Zeiss LSM780 confocal microscope (Carl Zeiss, Oberkochen, Germany), using a Plan-Apochromat 40x/1.4 Oil DIC objective. The 3D reconstructions and measurements were done using the filament tracer wizard of Imaris 10.0 (Bitplane). Three cells per mouse were randomly selected for analysis and their values were averaged. Imaris-based quantification of microglia and astrocytes morphology was analyzed using 2-way ANOVA with Bonferroni’s post hoc test for multiple comparisons.

**Human CSF protein analysis**

CSF samples were collected by lumbar puncture, centrifuged, and stored at –80°C in polypropylene tubes. In order to classify participants in biomarker-related subgroups, Aβ42, p-tau and total tau (t-tau) levels were measured locally. For the EMIF-AD MBD and the Maastricht BB-ACL studies, INNOTEST enzyme-linked immunosorbent assays (ELISAs; Fujirebio; n=403) or Alzbio3 xMAP Luminex (n=91) techniques were used for those targeted measures. For the Washington University Knight ADRC cohort, levels of Aβ40, Aβ42, t-tau, p-tau were measured locally by chemiluminescent enzyme immunoassay using LUMIPULSE G1200 (Fujirebio, Malvern, PA). For the proteomic analysis, CSF samples were shipped on dry ice to the Neurochemistry Lab of University of Gothenburg in Mölndal, Sweden. Untargeted central proteomic and peptidomic analyses were performed using the tandem mass tag (TMT) technique, as described elsewhere (2-4). A total of 3102 proteins were quantified. For further analysis, only the proteins that had at least one third of observations per participant group were included. All analyses were performed according to the manufacturer's instructions in two occasions (one batch for Maastricht BB-ACL and EMIF-AD MBD cohorts and one batch for Washington University ADRC and Antwerp cohorts) by board-certified laboratory technicians who were blinded to clinical information.

**Participant classification**

Participants were classified based on their cognitive status, as well as their CSF levels of Aβ42 (A) and p-tau (T). A neuropsychological assessment was administered to all participants, including the Mini-Mental State Examination (MMSE) and neuropsychological tests assessing several cognitive domains. Neuropsychological tests differed between centers but most common tests were the Rey Auditory Verbal Learning Test (for BB-ACL and EMIF-AD MBD studies) and the Free and Cued Selective Reminding Test (for Washington University Knight ADRC study) to assess memory, the Trail Making Test A for attention, the Trail Making Test B for executive functioning and Animal Fluency for language. Detailed information about the neuropsychological tests and calculation of Z-scores can be found elsewhere (5-7). Cognition was defined as normal if neuropsychological test performance ranged within 1.5 standard deviations (SDs) of the average corrected for age, sex, and education. Diagnosis of MCI was according to the criteria of Petersen (5, 8). Diagnosis of AD dementia was made using the the National Institute of Neurological and Communicative Disorders and Stroke–Alzheimer’s Disease and Related Disorders Association criteria (NINCDS-ADRDA) criteria (5, 9). We used cohort-specific cut-offs to define abnormal biomarker levels (2, 10). As center-specific methodologies were used to determine Aβ42 cut-offs, those were redefined for each cohort using unbiased Gaussian mixture modelling (Additional Table 1) (11).

**Additional Results**

**Characteristics of the APP^NL-G-F^ mouse model**

To test the viability of our mouse model, we evaluated the amyloidosis, microgliosis and astrocytosis in the APP^NL-G-F^ mice (Additional Figure 1). Amyloid-positive plaques were present in the hippocampus of the APP^NL-G-F^ mice, mainly at 40 weeks old, while absent in controls (Additional Figure 1A). Microgliosis was found in the hippocampus of the 7 weeks old APP^NL-G-F^ mice compared to their respective controls, with significant smaller microglial processes. A trend could also be observed for the other examined microglial parameters. No significant microglial changed were observed between the 40 weeks old APP^NL-G-F^ mice and their respective WT (Additional Figure 1B-C). Astrocytosis was found in the hippocampus of the 7 and 40 weeks old APP^NL-G-F^ mice compared to their respective controls. Indeed, astrocytes showed a significant increase in the number of segments, branches and terminal points, higher processes and volume in the APP^NL-G-F^ mice. Interestingly, the 40 weeks old APP^NL-G-F^ mice showed more activated astrocytes compared with the 7 weeks old APP^NL-G-F^ mice. The same observations were done for the WT mice (Additional Figure 1D-E).

**CSF proteomic profile of the APP^NL-G-F^ mouse model**

The 7 weeks old APP^NL-G-F^ mice showed 14 decreased and 34 increased proteins compared to their respective wild-type mice (Figure 2A, Additional Table 3). The decreased proteins were associated with pathways linked with vascular system, actin, ECM, immune system (related to macrophages, complement), oxidative stress, lipids and protein processing (Figure 2B-C). Out of the 14 decreased proteins, 13 had a role in ChP functioning, as they were also present in the ChP proteomic dataset. Five were highly expressed by the ChP (Figure 2A) and were associated with ECM, lipids and oxidative stress. The increased proteins were related to pathways associated with the extracellular matrix (ECM), phagosome, immune system (related to neutrophils, macrophages, B cells and immunoglobulins), lysosome and protein modification (Figure 2D-E). Out of the 34 increased proteins, 32 had a role in ChP functioning. Twelve were highly expressed by the ChP (Figure 2A) and were related to lysosomes, ECM, lipids and actin.

The CSF proteome of the 40 weeks old APP^NL-G-F^ mice showed 15 decreased and 5 increased proteins compared to the matched control mice (Figure 2F, Additional Table 3). The decreased proteins were associated with pathways linked with the vascular system and insulin, ECM and endocytosis (Figure 2G-H). Out of the 15 decreased proteins, 14 had a role in ChP functioning. Four were highly expressed by the ChP (Figure 2F) and were associated with ECM and endocytosis. The increased proteins were related to pathways associated with coagulation (Figure 2I-J). All the increased proteins had a role in ChP functioning, as they were also present in the ChP proteomic dataset. One out of the 5 increased proteins was highly expressed by the ChP (Figure 2F) and related to lipids.

Comparing the significant CSF dysregulated proteins in both the 7 and 40 weeks comparisons, we found an overlap in only 8 proteins (Additional Table 3), of which 7 had a role in ChP functioning and 3 were highly expressed by the ChP. Most of the implicated pathways were also different between the 7 and 40 weeks comparisons. Nonetheless, at both ages, ChP-associated pathways linked with ECM were dysregulated.

**CSF proteomic profile of the human participants**

In NC A+T-, 94 proteins were decreased and 55 proteins were increased compared to the controls (Figure 3A). Out of the 94 decreased proteins, 16 were highly expressed by the ChP and were linked with immune system (related to NK cells, leukocytes and macrophages) and vascular system (Figure 3B-C). Out of the 55 increased proteins, 31 were highly expressed by the ChP and were associated with lysosomes, vascular system, oxidative stress, and ECM (Figure 3D-E).

In MCI A+T-, 330 proteins were decreased and 120 proteins were increased compared to the controls (Figure 3F). Out of the 330 decreased proteins, 53 were highly expressed by the ChP and were linked with immune system (related to the complement and antigen presentation), vascular system, lipids, energy metabolism and mitochondria, oxidative stress, protein processing, and ECM (Figure 3G-H). Out of the 120 increased proteins, 46 were highly expressed by the ChP and were associated with vascular system, ECM, lysosomes and protein degradation (Figure 3I-J).

In AD A+T-, 379 proteins were decreased and 49 proteins were increased compared to the controls (Additional Figure 2A). Out of the 379 decreased proteins, 57 were highly expressed by the ChP and were linked with immune system (related to antigen presentation), oxidative stress, lipids, epithelial cells, vascular system, ECM, protein processing, and energy metabolism and mitochondria (Additional Figure 2B-C). Out of the 49 increased proteins, 16 were highly expressed by the ChP and were associated with immune system (linked with cytokines and interleukins), vascular system, and protein degradation (Additional Figure 2D-E).

In post-hoc analysis, we corrected our analysis for batch effects, which resulted in similar findings.

**References**

1. Chiva C, Olivella R, Borras E, Espadas G, Pastor O, Sole A, et al. QCloud: A cloud-based quality control system for mass spectrometry-based proteomics laboratories. PLoS One. 2018;13(1):e0189209. Epub 2018/01/13. doi: 10.1371/journal.pone.0189209. PubMed PMID: 29324744; PubMed Central PMCID: PMCPMC5764250.

2. Delvenne A, Gobom J, Tijms B, Bos I, Reus LM, Dobricic V, et al. Cerebrospinal fluid proteomic profiling of individuals with mild cognitive impairment and suspected non-Alzheimer's disease pathophysiology. Alzheimers Dement. 2022. Epub 2022/06/15. doi: 10.1002/alz.12713. PubMed PMID: 35698882.

3. Batth TS, Francavilla C, Olsen JV. Off-line high-pH reversed-phase fractionation for in-depth phosphoproteomics. J Proteome Res. 2014;13(12):6176-86. Epub 2014/10/23. doi: 10.1021/pr500893m. PubMed PMID: 25338131.

4. Magdalinou NK, Noyce AJ, Pinto R, Lindstrom E, Holmen-Larsson J, Holtta M, et al. Identification of candidate cerebrospinal fluid biomarkers in parkinsonism using quantitative proteomics. Parkinsonism Relat Disord. 2017;37:65-71. Epub 2017/02/19. doi: 10.1016/j.parkreldis.2017.01.016. PubMed PMID: 28214264.

5. Bos I, Vos S, Vandenberghe R, Scheltens P, Engelborghs S, Frisoni G, et al. The EMIF-AD Multimodal Biomarker Discovery study: design, methods and cohort characteristics. Alzheimers Res Ther. 2018;10(1):64. Epub 2018/07/08. doi: 10.1186/s13195-018-0396-5. PubMed PMID: 29980228; PubMed Central PMCID: PMCPMC6035398.

6. Grober E, Petersen KK, Lipton RB, Hassenstab J, Morris JC, Gordon BA, et al. Association of Stages of Objective Memory Impairment With Incident Symptomatic Cognitive Impairment in Cognitively Normal Individuals. Neurology. 2023;100(22):e2279-e89. Epub 2023/04/20. doi: 10.1212/WNL.0000000000207276. PubMed PMID: 37076305; PubMed Central PMCID: PMCPMC10259282 Selective Reminding Test with Immediate Recall (FCSRT + IR). The test is available at no cost to researchers and clinicians. The Albert Einstein College of Medicines holds the copyright for the test. Inquiries should be addressed to Dr. Nilam Sinha in the Office of Biotechnology and Business Development (Nilam.Sinha@einsteinmed.org). She is a paid consultant for Genentech-Roche. K.K. Petersen reports no disclosures relevant to the manuscript. R.B. Lipton serves as a consultant and advisory board member or has received honoraria for his headache work from AbbVie (Allergan), the American Academy of Neurology, the American Headache Society, Amgen, Biohaven, Eli Lilly, GlaxoSmithKline, Grifols, Lundbeck (Alder), Merck, Pfizer, Teva, Vector, and Vedanta. He receives royalties from Wolff's Headache 7th and 8th Edition, Oxford Press University, 2009, Wiley and Informa. J. Hassenstab, J.C. Morris, B. Gordon, and A.Ezzati report no disclosures relevant to the manuscript. Go to Neurology.org/N for full disclosures.

7. Bos I, Verhey FR, Ramakers I, Jacobs HIL, Soininen H, Freund-Levi Y, et al. Cerebrovascular and amyloid pathology in predementia stages: the relationship with neurodegeneration and cognitive decline. Alzheimers Res Ther. 2017;9(1):101. Epub 2017/12/30. doi: 10.1186/s13195-017-0328-9. PubMed PMID: 29284531; PubMed Central PMCID: PMCPMC5747152.

8. Petersen RC. Mild cognitive impairment as a diagnostic entity. J Intern Med. 2004;256(3):183-94. Epub 2004/08/25. doi: 10.1111/j.1365-2796.2004.01388.x. PubMed PMID: 15324362.

9. McKhann G, Drachman D, Folstein M, Katzman R, Price D, Stadlan EM. Clinical diagnosis of Alzheimer's disease: report of the NINCDS-ADRDA Work Group under the auspices of Department of Health and Human Services Task Force on Alzheimer's Disease. Neurology. 1984;34(7):939-44. Epub 1984/07/01. doi: 10.1212/wnl.34.7.939. PubMed PMID: 6610841.

10. Morris JC, Schindler SE, McCue LM, Moulder KL, Benzinger TLS, Cruchaga C, et al. Assessment of Racial Disparities in Biomarkers for Alzheimer Disease. JAMA Neurol. 2019;76(3):264-73. Epub 2019/01/08. doi: 10.1001/jamaneurol.2018.4249. PubMed PMID: 30615028; PubMed Central PMCID: PMCPMC6439726.

11. Tijms BM, Willemse EAJ, Zwan MD, Mulder SD, Visser PJ, van Berckel BNM, et al. Unbiased Approach to Counteract Upward Drift in Cerebrospinal Fluid Amyloid-beta 1-42 Analysis Results. Clin Chem. 2018;64(3):576-85. Epub 2017/12/07. doi: 10.1373/clinchem.2017.281055. PubMed PMID: 29208658.

**Tables and Figures**

Additional Table 1. Center-specific Aβ42 and p-tau cut-off point.

Additional Table 2. Dysregulated proteins for each comparison in the whole list of identified proteins in mouse ChP tissue.

Additional Table 3. Dysregulated proteins for each comparison in the whole list of identified proteins in mouse CSF.

Additional Table 4. Dysregulated proteins for each comparison in the whole list of identified proteins in human CSF.

Additional Figure 1. Amyloid deposition and astrocytic and microglial characteristics of our AD mouse model (APP^NL-G-F^) and their relative wild-type (WT).

Additional Figure 2. CSF proteomic profiles and dysregulated pathways in AD dementia A+T-.

Additional Figure 3. Similar dysregulated proteins highly expressed by the ChP in NC A+T-, MCI A+T- and AD dementia A+T-.

Additional Figure 4. CSF proteomic profiles of NC A+T+, MCI A+T+ and AD dementia A+T+.

**Tables:**

| **Additional Table 1. Center-specific Aβ42 and p-tau cut-off point.** | | |
| --- | --- | --- |
| **Centers** | **cut-off CSF Aβ42 (pg/ml)** | **cut-off CSF p-tau (pg/ml)** |
| EMIF-AD MBD | | |
| Amsterdam [1] | *<813* | *>52* |
| Antwerp [2] | *<669* | *>56.5* |
| DESCRIPA [3] | *<530* | *>52* |
| EDAR [4]* | *<316* | *>35* |
| GAP [5] | *<655* | *>61* |
| Lausanne [6] | *<752* | *>55* |
| Leuven [7] | *<626* | *>80* |
|  |  |  |
| Maastricht [8] | *<775* | *>52* |
|  |  |  |
| WashU Knight ADRC [9] | *<588* | *>44.3* |
| **EDAR used Luminex assay and the other centers used ELISA. References are given for each center. Abbreviations: CSF = cerebrospinal fluid, Aβ42 = amyloid beta, p-tau = phosphorylated tau*  *References: [1] van der Flier, W.M., et al., Optimizing patient care and research: the Amsterdam Dementia Cohort. J Alzheimers Dis, 2014. 41(1): p. 313-27.; [2] Somers C, Struyfs H, Goossens J, Niemantsverdriet E, Luyckx J, De Roeck N, De Roeck E, De Vil B, Cras P, Martin JJ, De Deyn PP, Bjerke M, Engelborghs S. A Decade of Cerebrospinal Fluid Biomarkers for Alzheimer's Disease in Belgium. J Alzheimers Dis. 2016 Aug 10;54(1):383-95. doi: 10.3233/JAD-151097. PMID: 27567807. [3] Visser, P.J., et al., Development of screening guidelines and clinical criteria for predementia Alzheimer's disease. The DESCRIPA Study. Neuroepidemiology, 2008. 30(4): p. 254-65.; [4] Reijs, B.L.R., et al., Relation of Odor Identification with Alzheimer's Disease Markers in Cerebrospinal Fluid and Cognition. J Alzheimers Dis, 2017. 60(3): p. 1025-1034.; [5] Estanga, A., et al., Beneficial effect of bilingualism on Alzheimer's disease CSF biomarkers and cognition. Neurobiol Aging, 2017. 50: p. 144-151.; [6] Tautvydaite, D., et al., Interaction between personality traits and cerebrospinal fluid biomarkers of Alzheimer's disease pathology modulates cognitive performance. Alzheimers Res Ther, 2017. 9(1): p. 6.; [7] Adamczuk, K., et al., Amyloid imaging in cognitively normal older adults: comparison between (18)F-flutemetamol and (11)C-Pittsburgh compound B. Eur J Nucl Med Mol Imaging, 2016. 43(1): p. 142-151. [8] Bos, I., et al., Cerebrovascular and amyloid pathology in predementia stages: the relationship with neurodegeneration and cognitive decline. Alzheimers Res Ther, 2017. 9(1): p. 101. [9]* *Volluz KE, Schindler SE, Henson RL, et al. Correspondence of CSF biomarkers measured by Lumipulse assays with amyloid PET. Alzheimer's & Dementia. 2021;17(S5):e051085.* | | |

**Additional Table 2. Dysregulated proteins for each comparison in the whole list of identified proteins in mouse ChP tissue.** List of all the identified proteins in mouse ChP tissue using a threshold of 3 observations per group minimum. The significant p values and the log2 fold changes are shown for each comparison.

**Additional Table 3. Dysregulated proteins for each comparison in the whole list of identified proteins in mouse CSF.** List of all the identified proteins in mouse CSF using a threshold of 3 observations per group minimum. The significant p values and the log2 fold changes are shown for each comparison.

**Additional Table 4. Dysregulated proteins for each comparison in the whole list of identified proteins in human CSF.** List of all the human proteins (A+T- groups) using a threshold of 1/3 observations per group minimum. The analyses were adjusted for age and gender. The significant p values and the direction of the level changes are shown for each comparison. The proteins with a high expression in the ChP are shown.

**Figures:**

**Additional Figure 1.** Amyloid deposition and astrocytic and microglial characteristics of our AD mouse model (APP^NL-G-F^) and their relative wild-type (WT).


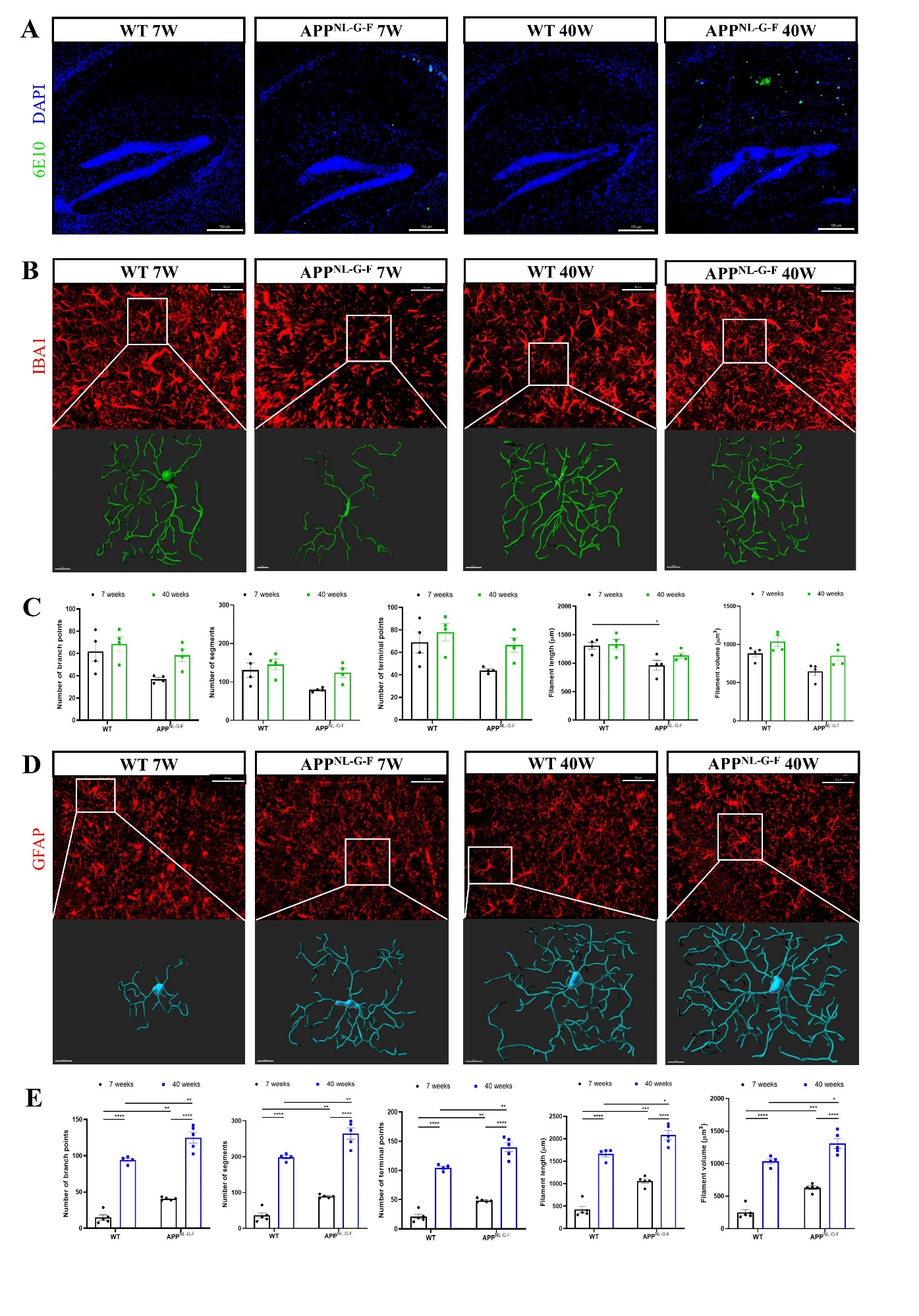


(A) Representative images of 6E10 staining in hippocampus showing the presence of amyloid-positive plaques (arrows) in APP^NL-G-F^ mice, which are absent in controls. Scale bar: 150 μm. (B) 3D surface reconstruction images of IBA1+ microglial cells from hippocampus generated from confocal microscopic imaging using Imaris software. Scale bar: 50 μm. (C) Imaris-based quantification of morphological parameters of IBA1+ microglial cells in hippocampus. Each symbol represents one mouse, 3 cells per mouse were randomly selected for analysis and their values were averaged (n = 4–5). Mean ± SEM, two-way ANOVA Bonferroni’s post hoc test for multiple comparisons. * = 0.01≤p<0.05 (D) 3D surface reconstruction images of GFAP+ astrocytic cells from hippocampus generated from confocal microscopic imaging using Imaris software. Scale bar: 50 μm. (E) Imaris-based quantification of morphological parameters of GFAP+ astrocytic cells in hippocampus. Each symbol represents one mouse, 3 cells per mouse were randomly selected for analysis and their values were averaged (n = 4–5). Mean ± SEM, two-way ANOVA Bonferroni’s post hoc test for multiple comparisons. * = 0.01≤p<0.05; ** = 0.001≤ p<0.01; *** = 0.0001≤p<0.001; ****= p<0.0001.

**Additional Figure 2. CSF proteomic profile and associated pathways in AD dementia A+T-.**


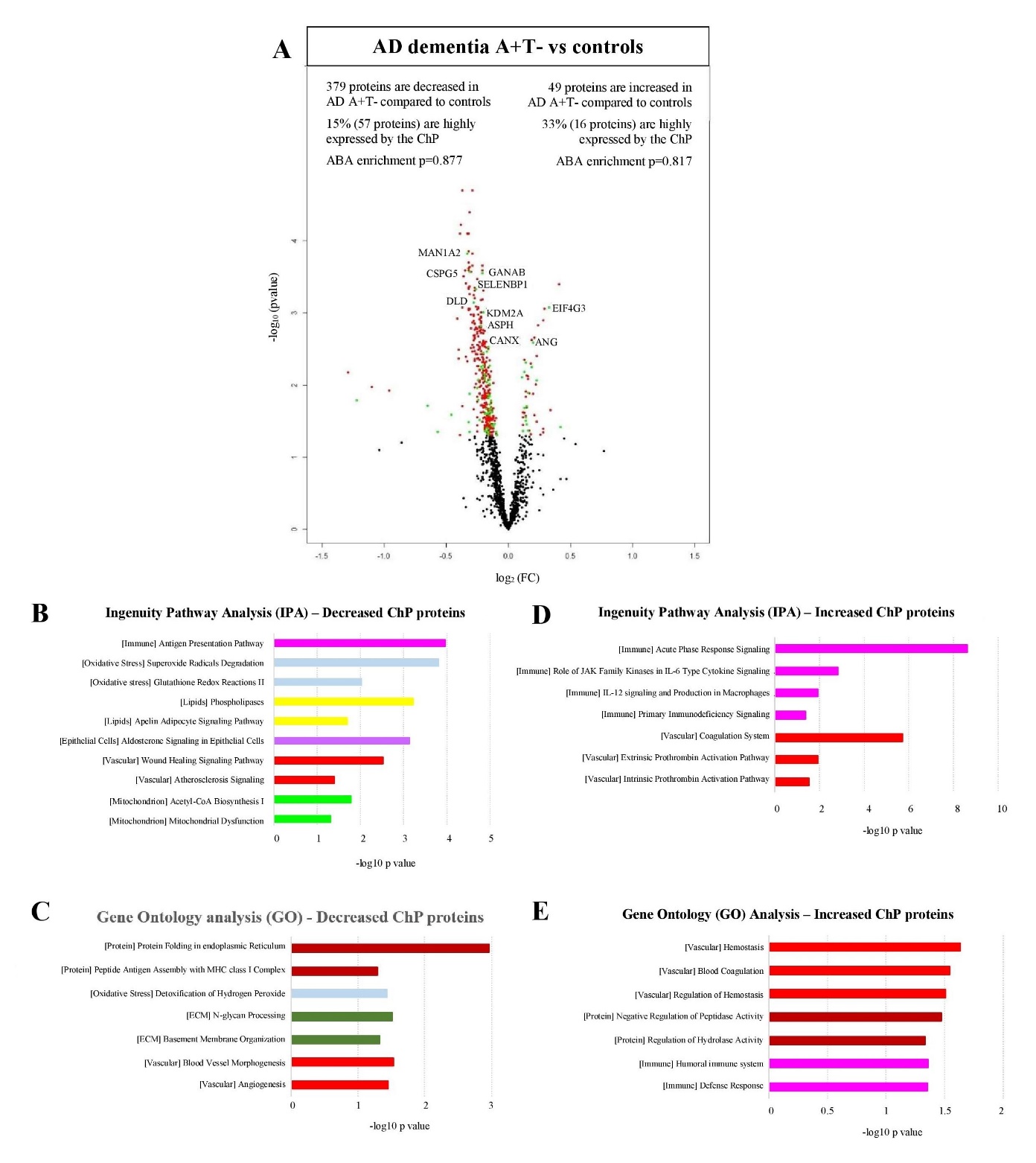


(A) Volcano plot displaying the log2 fold-change against the -log10 statistical P-value for the comparison AD dementia A+T- vs controls. Significantly different proteins are red. Significantly different proteins highly expressed by the ChP are green. The top 10 proteins highly expressed by the ChP are named. The number of proteins highly expressed by the ChP, as well as the gene expression enrichment in the ChP (ABAenrichment) p-value, are displayed. (B) Selected canonical pathways from Ingenuity pathway analysis (IPA) for the decreased proteins highly expressed by the ChP in the comparison AD dementia A+T- vs controls. (C) Selected Gene Ontology (GO) terms including biological process for the decreased proteins highly expressed by the ChP in the comparison AD dementia A+T- vs controls. (D) Selected canonical pathways from IPA for the increased proteins highly expressed by the ChP in the comparison AD dementia A+T- vs controls. (E) Selected GO terms including biological process for the increased proteins highly expressed by the ChP in the comparison AD dementia A+T- vs controls. Immune-related pathways are pink, pathways associated with oxidative stress are light blue, pathways linked with lipids are yellow, epithelial cells related pathways are light purple, vascular-related pathways are in red, pathways related to energy metabolism and mitochondria are light green, protein-linked pathways are brown, pathways related to ECM are dark green.

**Additional Figure 3.** **Similar dysregulated proteins highly expressed by the ChP in NC A+T-, MCI A+T- and AD dementia A+T-.**


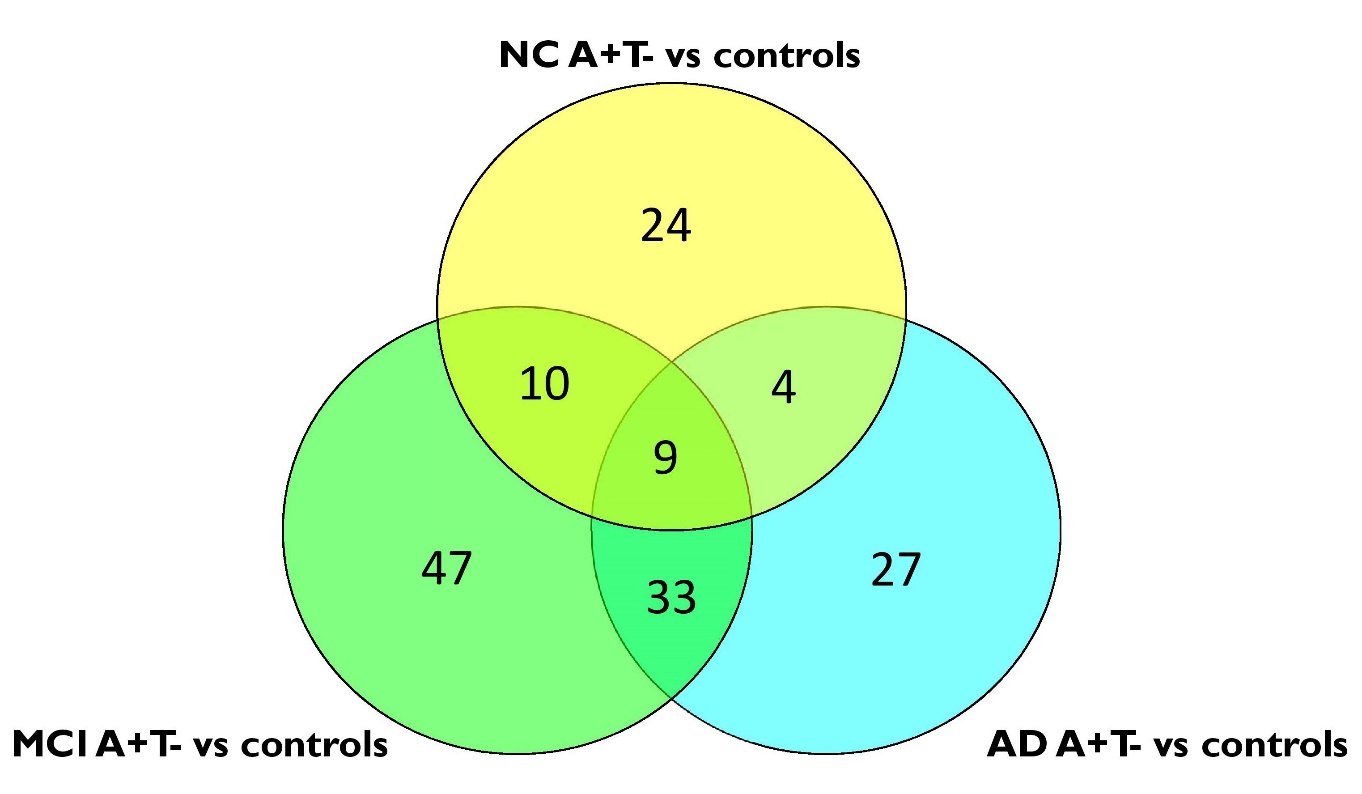


Venn diagram representing the significant proteins highly expressed by the ChP for each comparison.

**Additional Figure 4. CSF proteomic profiles of NC A+T+, MCI A+T+ and AD dementia A+T+.**


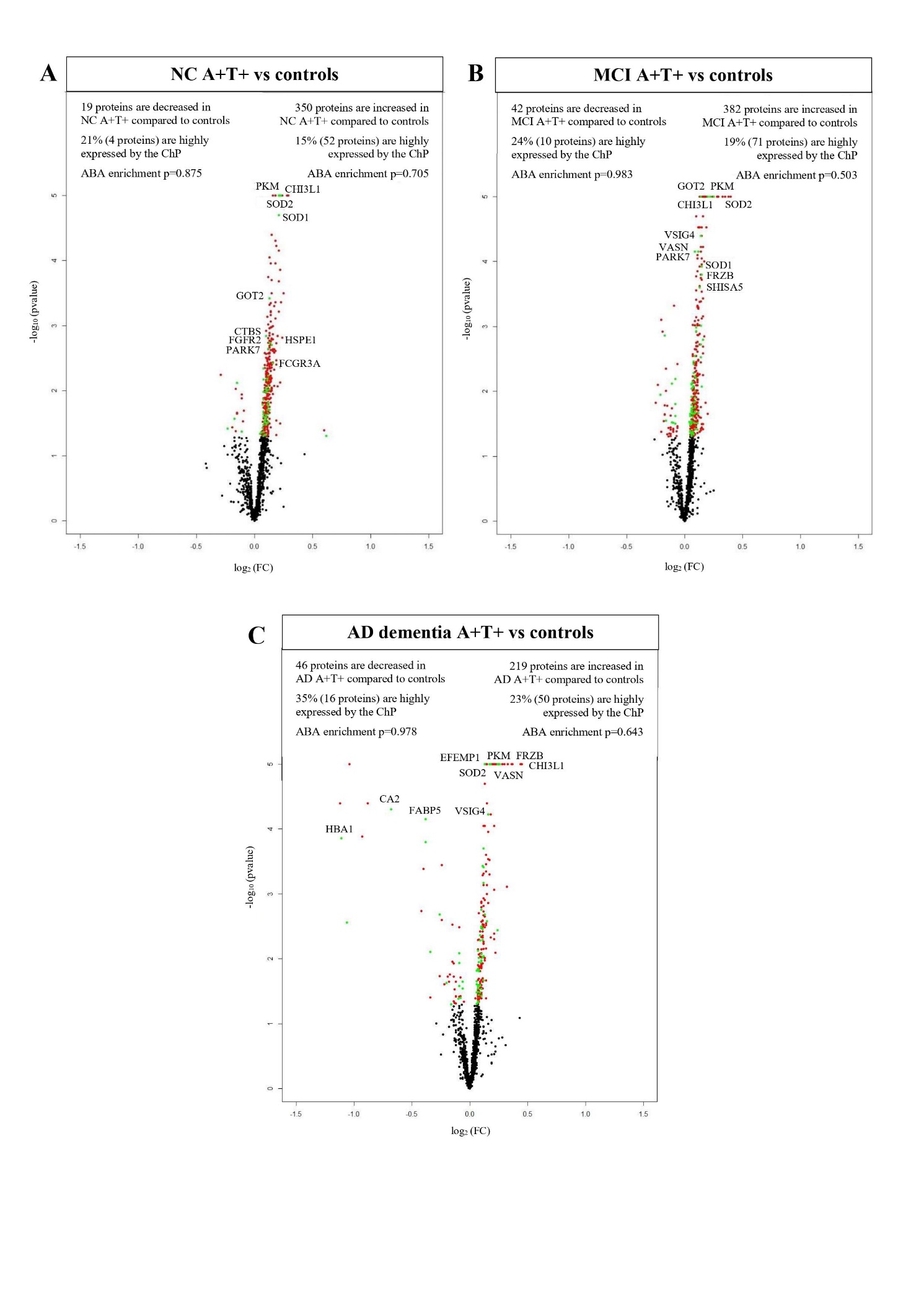


(A) Volcano plot displaying the log2 fold-change against the -log10 statistical P-value for the comparison NC A+T+ vs controls. Significantly different proteins are red. Significantly different proteins highly expressed by the ChP are green. The top 10 proteins highly expressed by the ChP are named. The number of proteins highly expressed by the ChP, as well as the gene expression enrichment in the ChP (ABAenrichment) p-value, are displayed. (B) Volcano plot displaying the log2 fold-change against the -log10 statistical P-value for the comparison MCI A+T+ vs controls. Significantly different proteins are red. Significantly different proteins highly expressed by the ChP are green. The top 10 proteins highly expressed by the ChP are named. The number of proteins highly expressed by the ChP, as well as the gene expression enrichment in the ChP (ABAenrichment) p-value, are displayed. (C) Volcano plot displaying the log2 fold-change against the -log10 statistical P-value for the comparison AD dementia A+T+ vs controls. Significantly different proteins are red. Significantly different proteins highly expressed by the ChP are green. The top 10 proteins highly expressed by the ChP are named. The number of proteins highly expressed by the ChP, as well as the gene expression enrichment in the ChP (ABAenrichment) p-value, are displayed.
